# Headspace Solid-Phase Microextraction and Gas Chromatography–Mass Spectrometry Combined with Sensory Evaluation for the Analysis of Volatile Aromatic Compounds in Apricot (*Prunus armeniaca* L.) Germplasm Resources Cultivated in Xinjiang, China

**DOI:** 10.3390/foods13233912

**Published:** 2024-12-03

**Authors:** Xueling Zeng, Shikui Zhang, Wenjuan Geng, Jie Jin, Kang Liao, Zhanghu Tang, Shaopeng Wang, Weiquan Zhou

**Affiliations:** 1 College of Horticulture, Xinjiang Agricultural University, Urumqi 830052, China; xuelingz0118@163.com (X.Z.); gwj0526@xjau.edu.cn (W.G.); 17767622134@163.com (J.J.); liaokang01@163.com (K.L.); 19915127905@163.com (S.W.); 2 Luntai Fruit Germplasm Resources Garden, Xinjiang Academy of Agricultural Sciences, Luntai 841600, China; zsk2366287@163.com (S.Z.); tangzhanghu@163.com (Z.T.); 3 Postdoctoral Mobile Station of Horticulture, College of Horticulture, Xinjiang Agricultural University, Urumqi 830052, China

**Keywords:** apricot, aromatic components, relative odor activity value, volatile metabolomics, gas chromatography–mass spectrometry

## Abstract

The volatile compounds in the fruits of 24 apricot cultivars were quantitatively and qualitatively determined via headspace solid-phase microextraction and gas chromatography–mass spectrometry (HS-SPME-GC–MS). A total of 429 volatile compounds were detected in these fruits, and the greatest number of detected terpenoids was 77. Significant differences were found among the cultivars in terms of the total volatile compound content of the fruits, with variation from 112.76 (‘ZSHYX’) to 317.36 µg/g (‘JNL’). Using relative odor activity value (rOAV) analysis, 42 key aroma compounds were identified. The rOAVs of (2S,4R)-4-methyl-2-(2-methylprop-1-enyl)oxane, (E)-non-2-enal, (3-methyl-3-sulfanylbutyl) formate, and thiophen-2-ylmethanethiol were above 1000, and most had green, fruity, and woody odors; these results indicated that these substances were important contributors to the overall aroma of the apricot fruits. Our study provides a comprehensive analysis of the volatile compounds from 24 representative apricot cultivars and can aid in the further scientific understanding of the metabolites and aroma in apricots. These findings provide a reference for controlling fruit quality and for future apricot cultivar breeding.

## 1. Introduction

In higher plants, volatile compounds are widely present and important secondary metabolites [1]. Volatile compounds are crucial in the interactions between plants and their surroundings because they attract pollinators and seed dispersers. Plants resist and adapt to their external environment by synthesizing and releasing volatile compounds, thereby protecting themselves from parasites, herbivores, and pathogens [2]. In addition, volatile compounds are a source of olfactory stimulation in humans; they provide aromas to cereals, fruits, and vegetables and have a large impact on flavoring, preservatives, and herbal remedies [3,4]. Aroma is one of the most important organoleptic characteristics of fruits; volatile compounds constitute only 10^−4^ to 10^−7^ of the weight of fresh fruit and are secondary metabolites formed from terpenoids, esters, heterocyclic compounds, and alcohols [5,6]. Aroma is a crucial factor influencing consumer perception and acceptance of fruit products and has a great impact on appetite and the digestive system, which are important for transmitting nutritional signals to humans and can even affect a person’s mental state [7,8]. Researchers have increasingly shown great interest in fruit aroma.

The apricot species *Prunus armeniaca* L. is a widely cultivated drupe fruit tree, and Italy, Turkey, the United States, Spain, France, and China are the biggest apricot-producing regions of the world [9]. Apricots are highly appreciated by consumers for their rich nutritional content and unique aroma, and they can be eaten raw or dried and processed [10]. The earliest research on the aroma compounds of apricot fruits was conducted in 1967, and Tang and Jenning were the first to identify and analyze volatile compounds from apricot fruits in a European ecosystem [11]. Many scholars have extensively researched the components of volatile compounds in apricot fruits. Solís-Solís et al. [12] studied the aroma-imparting substances of the fruits of eight apricot varieties via four extraction techniques, and their results revealed that the extraction technique and the variety had large influences on the fruit aroma type and content. Gas chromatography–mass spectrometry (GC–MS) can be used to comprehensively identify volatile compounds in fruits. Zhang et al. [13] analyzed the volatile profiles of the fruits of apricot germplasm resources in different regions of China and reported that the contents and components of volatile profiles in these fruits greatly varied. The volatile compound content was the highest for apricot varieties in Northwest China but was low for apricot varieties in Southwest China. These results revealed that genetic background strongly affected the contents and components of volatile compounds. Using an odor activity value (OAV) analysis, Greger and Schieberle [14] reported that (Z)-1,5-octadien-3-one, β-ionone, γ-decalactone, acetaldehyde, linalool, and (E, Z)-2,6-nonadienal had OAVs greater than 100 and were key aroma components of apricot fruit.

Headspace solid-phase microextraction (HS-SPME) is a simple procedure that has a low monitoring threshold, good detection sensitivity, and good repeatability. This technique can be combined with gas chromatography–mass spectrometry (GC–MS) to obtain more comprehensive and reliable data. It can be used to identify, simultaneously separate, and provide specific flavor information and quantify multiple volatile compounds [15,16]. Although many reports have described the contents and components of volatile compounds in apricot fruits, no systematic studies have been conducted on the composition and content of volatile compounds in Xinjiang apricot fruits, and the use of volatile metabolite analysis to qualitatively and quantitatively evaluate volatile compounds in apricot fruits has not been reported. In this study, sensory analysis combined with HS-SPME-GC–MS was used to qualitatively and quantitatively analyze and compare the volatile compounds in the fruits of 24 apricot cultivars, and key aroma compounds were identified via the relative odor activity value (rOAV) method. The aim of this study was to explore aroma information to understand the quality differences among a wider range of apricot germplasm resources and provide a reference for apricot fruit breeding and quality control in the future. In addition, these results provide valuable data on apricot aroma for future research on genes that regulate aroma components in combination with genetic linkage mapping and genome-wide association study (GWAS) analysis.

## 2. Materials and Methods

### 2.1. Sample Preparation and Methods for Determining the Fruit Quality Index

The samples were collected from the Luntai Fruit Germplasm Resources Garden of the Xinjiang Academy of Agricultural Sciences (E 84°13′, N 41°47′). The experimental site was in the Luntai County, Bayingoleng Mongolian Autonomous Prefecture, Xinjiang Uygur Autonomous Region, China, and pictures of the mature fruits of the 24 apricot cultivars are shown in Supplementary Figure S1. Conventional water and fertilizer management practices were employed. From June to July 2023, samples were collected from the fruits of each cultivar during the ripening period, and the criteria for determining the ripening period of the fruits were their hardness, color, and soluble solid content. Three apricot trees of each variety were randomly selected, and 100 apricot fruits were collected from the outer canopy. The cultivar samples were randomly divided into three parts. Each sample was quickly cut into small pieces with a scalpel, frozen, and submerged in liquid nitrogen. After mixing, the apricot samples were immediately placed in a frozen tube and stored at −80 °C to determine the volatile compounds. The other portion was used for quality index determination. The longitudinal diameter, transverse diameter, and lateral diameter of the apricot fruit were measured with a digital display caliper, an electronic balance (MP2001, Shanghai, China) was used to measure the fruit weight (g), and a portable pH meter (Horiba, Kyoto, Japan) was used to measure the pH of the apricot fruit. Fruit hardness was determined with a hardness tester (GY-4, Zhejiang, China). The soluble solids were measured with a portable refractometer (Atago, Tokyo, Japan). The edible rate (pitted fresh fruit quality as a percentage of the total fresh fruit quality) and fruit water content (dried fruit quality as a percentage of fresh fruit quality) were also determined.

### 2.2. Panel Training and Aroma Characteristic Analysis

The sensory properties of the fruits were evaluated according to the methods of Fan et al. [17]. The sensory assessment team consisted of four female members and four male members who were trained in sensory assessment. Sweet, green, fruity, floral, herbal, woody, waxy, fatty, citrusy, and nutty aromas were evaluated. The sensory evaluation was graded on a 1–60 scale, with 1 representing very low strength and 60 representing the highest strength. Ten fruits were tested for each apricot cultivar, and the average of all scores was calculated.

### 2.3. Volatile Extraction and Concentration

The extraction steps were as follows: the apricot frozen sample was removed from storage at ultralow temperatures, 500 mg of sample was weighed, −80 °C liquid nitrogen was added, and the sample was ground to powder. Then, 500 mg powder sample was weighed and dissolved in 2 mL saturated NaCl solution (analytical reagent). A 20 µL internal standard solution consisting of 3-hexanone-2, 2, 4, 4-d4 (10 μg/mL) was added, the sample was promptly transferred to an Agilent USA 20 mL headspace vial, and the addition of a saturated NaCl solution inhibited any enzyme reactions. In this study, an SPME extractor with 120 μm DVB/CWR/PDMS (Agilent, Santa Clara, CA, USA) was used with an SPME Arrow (CTC Analytics AG, Basel, Switzerland). The extractor was set to 250 °C for 5 min before use. A rolled cap with a TFE silicone headspace diaphragm (Agilent) was used to seal the vial. First, the headspace vial was shaken at 60 °C for 5 min; afterward, the aging extraction head was placed in the vial for 15 min. A gas chromatograph (Type 7890B, Agilent) injector was used for desorption at 250 °C for 5 min in nonshunt mode, and the sample was then removed [18].

### 2.4. Gas Chromatography–Mass Spectrometry Analyses

The test instrument used was a 7890B (Agilent) type gas chromatograph and 7000D mass spectrometer (Agilent). The column was a 30 m × 25 mm × 0.25 μm DB5-ms column (Agilent, Santa Clara, CA, USA). The constant flow rate of the high-purity carrier gas was 1.2 mL/min, the injection mode was splitless, and the inlet temperature was maintained at 250 °C. Initially, the temperature of the column was 40 °C, held for 3.5 min, then increased to 100 °C at 10 °C per minute, increased to 180 °C at 7 °C per minute, and finally increased to 280 °C at 25 °C per minute and held for 5 min. The mass spectrum conditions included an electron energy of 70 eV, a quadrupole mass detector temperature of 150 °C, an ion source temperature of 230 °C, and a mass spectrum interface temperature of 280 °C. Selective ion monitoring (SIM) was used as the scanning mode for mass spectrometry, the data were obtained via accurate qualitative and quantitative scanning, and the obtained mass spectra were compared with those in the MWGCSIM 1.0 library to complete the qualitative and quantitative analyses [19].

### 2.5. Qualitative and Quantitative Analysis

On the basis of the MWDB (MetWare database) 2023 Update of plant volatile substances, qualitative and quantitative analyses of the volatile substances in the cultivar samples were performed via mass spectrometry. Supplementary Figure S2 shows the total ion current (TIC) data of the samples. The standard substances used in this test were prepared by diluting n-hexane (chromatographic purity, CNW) to a certain concentration, and the solution was stored at −20 °C. The volatile substances in the cultivar samples were determined semiquantitatively via the internal standard method, and the relative contents of each volatile substance were determined using 3-hexanone-2, 2, 4, 4-d4 as the internal standard.

Regarding qualitative analysis, based on multiple species, the literature, partial reference standards, and retention indices, an independent database (the updated version of the MWGC database in 2023, provided by Wuhan Maiwei Metabolic Biotechnology Co., Ltd.) (Wuhan, China) was established, and an SIM mode detection protocol was constructed. All ions in each group were detected separately in time intervals according to the elution sequence. If the retention time of the detected chromatographic peak was consistent with that of the standard reference and if all selected ions appeared (2–3 qualitative fragments) in the sample mass spectrum after background subtraction, the sample was determined to be this substance. The qualitative method adopted in this study was supported by the existing methodologies used in the literature.

Quantitative analysis using Mass Hunter was conducted to increase the accuracy of the quantification. Specific quantification ions were carefully selected for integration and calibration. For the relative quantification of volatile compounds, the internal standard (3-hexanone-2,2,4,4-d4) semiquantitative method was employed. The resulting relative content of each volatile compound was as follows:

$$X_{i}=\frac{V_{s}\times C_{s}}{M}\times \frac{I_{i}}{I_{s}}\times 10^{-3}$$

where *X_i_* represents the content of compound *i* in the sample to be measured (μg/g); vs. represents the volume of the added internal standard (μL); *C_s_* represents the concentration of the internal standard (μg/mL); *M* is the amount of the sample to be measured (g); *I_s_* is the peak area of the internal standard; and *I_i_* is the peak area of compound *i* in the sample to be measured.

### 2.6. Volatile Compound Qualification and rOAV Calculation

The relative odor activity value (*rOAV*) is the ratio of the relative content of a volatile compound to its odor threshold (OT). Generally, compounds with an *rOAV* > 1 directly contribute to the aroma characteristics of fruits [20], and the *rOAV* content (μg/g) is determined via rOAVi=CiTi, where *rOAVi* is the relative odor activity of compound *i*, *Ci* is the relative content of compound *i* (μg/g), and *Ti* is the defined threshold of the compound (threshold, μg/g).

### 2.7. Statistical Analysis

Microsoft Excel 2020 and SPSS 23.0 were used for the statistical analysis of all three biological replicates. PCA (principal component analysis), PCC (Pearson correlation coefficient), and HCA (hierarchical cluster analysis) were performed using R language software 3.5.1.

## 3. Results

### 3.1. Analysis of the Quality Indices of Apricot Fruits at Maturity

The fruit quality characteristics of the different apricot cultivars are listed in Table 1. The maturity period of the 24 apricot cultivars ranged from 11 June to 11 July, and considerable differences in the single-fruit weight, pH, soluble solids, fruit shape index, water content, hardness, and edible rate were observed. The single-fruit weight varied among the cultivars and ranged from 18.52 to 100.91 g. The soluble solids ranged from 10.96 to 26.04%; here, the fruits of ‘MTYLK’ had the highest soluble solids, and those of ‘KBKYLK’ and ‘SGJNL’ had the lowest soluble solids. The fruit hardness ranged from 1.16 kg/cm^2^ to 6.44 kg/cm^2^. The fruit pH ranged from 3.63 to 5.10, and great differences were observed among the varieties. The fruit shape index ranged from 0.89 to 1.17, and the fruit shape index greatly varied among the different varieties. The fruit water content ranged from 73.17% to 91.73%. The variety with the highest edible rate was ‘ZGYDJX’, and the variety with the lowest edible rate was ‘DX’.

### 3.2. Analysis of the Sensory Flavor Characteristics of the Apricot Cultivars

**Figure 1:**
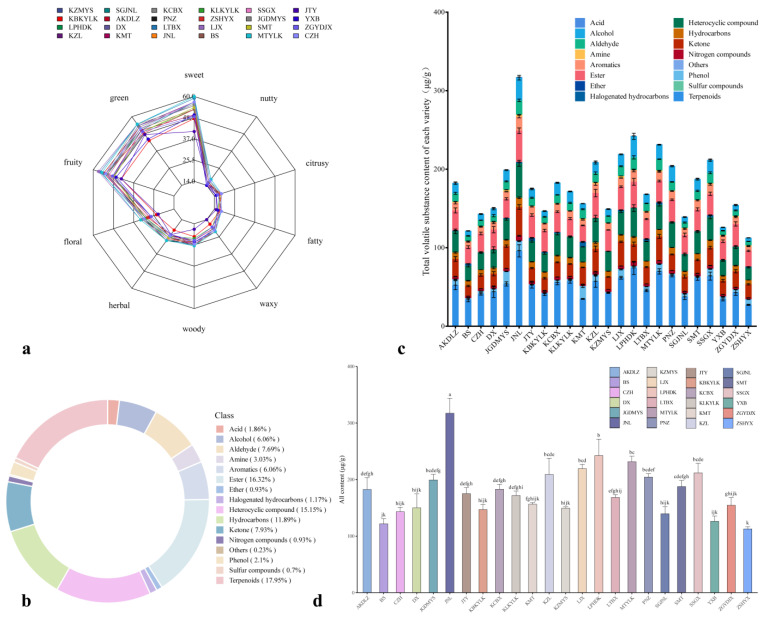
Analysis of the volatile compounds in apricot fruits: (**a**) Radar map of the European rich sensory flavor characteristics of 24 apricot cultivars; (**b**) volatile compound classes of the 24 apricot cultivars from a ring chart, where each color represents a metabolite class and the area of the color block represents the proportion of that class; (**c**) histogram of the metabolic analysis of the different types of volatile compounds in the fruits of 24 apricot cultivars; and (**d**) analysis of the difference in the total volatile compounds in the fruits of 24 apricot cultivars. A statistical analysis was performed via a one-way analysis of variance (ANOVA), and different letters indicate significant differences at the 0.05 level.

A group of professionals used the aroma sensory evaluation method to evaluate and analyze the aroma types of 24 apricot varieties and conducted a preliminary investigation and evaluation of the aroma intensity of different apricot varieties using sensory evaluation. These professionals agreed that all apricot fruits had recognizable sensory characteristics. The aroma types of the apricot fruits were classified as sweet, green, fruity, floral, herbal, woody, waxy, fatty, citrusy, and nutty (Figure 1a). However, the intensity of each type of aroma differed, and sweet, green, and fruity aromas were more prominent. The sensory results revealed that the scores of the ‘LJX’, ‘MTYLK’, ‘LTBX’, ‘AKDLZ’, ‘KZL’, and ‘KCBX’ cultivars were greater than 50, with scores of 57, 56, 56, 54, 54, and 54, respectively; these results indicated that these cultivars contained more volatile compounds.

### 3.3. Analysis of the Contents and Components of Volatile Compounds

The contents and components of volatile compounds in the fruits of 24 cultivars of apricot were quantitatively and qualitatively analyzed via HS-SPME-GC–MS. The contents and components of the volatile compounds in the apricots at maturity were compared (Supplementary Table S1). In this study, 429 volatile compounds were detected. The 429 volatile compounds were classified into 16 categories: terpenoids, heterocyclic compounds, esters, hydrocarbons, ketones, aldehydes, alcohols, aromatics, amines, phenols, acids, nitrogen compounds, halogenated hydrocarbons, ethers, sulfur compounds, and other unclassified compounds. Here, terpenoids were predominant, accounting for 77 species and 17.95% of all volatile compounds. In addition, 70 esters, 65 heterocyclic compounds, 51 hydrocarbons, 34 ketones, 33 aldehydes, 26 alcohols, and 26 aromatic compounds accounted for 16.32%, 15.15%, 11.89%, 7.93%, 7.69%, 6.06%, and 6.06%, respectively, of all volatile compounds. The above eight types of substances accounted for 89.05% of all the volatile compounds. The remaining volatile compounds included 13 amines, 9 phenols, 8 acids, 5 halogenated hydrocarbons, 4 nitrogen compounds, 4 ethers, 3 sulfur compounds, and 1 other unclassified compound. These compounds accounted for 3.03%, 2.1%, 1.86%, 1.17%, 0.93%, 0.93%, 0.7%, and 0.23% of all volatile compounds, respectively (Figure 1b). Among the 16 types of volatile compounds, terpenoids were the main volatile compounds in the 24 cultivars of apricot, followed by esters, heterocyclic compounds, hydrocarbons, ketones, and aldehydes; these compounds accounted for more than 70% of the total volatile compounds. The contents of the ether compounds, sulfur compounds, and other compounds in the fruits of the 24 apricot cultivars were less than 1.00 µg/g (Figure 1c). An analysis revealed a significant difference in the total content of volatile compounds in the fruits of the 24 apricot cultivars. The average total content of the aroma volatiles in these fruits was 179.37 ± 49.57 µg/g, and the range of volatile compound contents was 112.76 to 317.36 µg/g. The total content of the aromatic volatiles in the fruit of the ‘JNL’ cultivar was the highest (317.36 ± 26.36 µg/g), followed by ‘LPHDK’ and ‘MTYLK’ fruits (242.37 ± 29.07 µg/g and 231.63 ± 9.68 µg/g, respectively). ZSHYX had the lowest volatile compound content, at 112.76 ± 3.99 µg/g (Figure 1d).

### 3.4. Analysis of the Total Content of Volatile Compounds in the Fruits of 24 Apricot Cultivars

**Figure 2:**
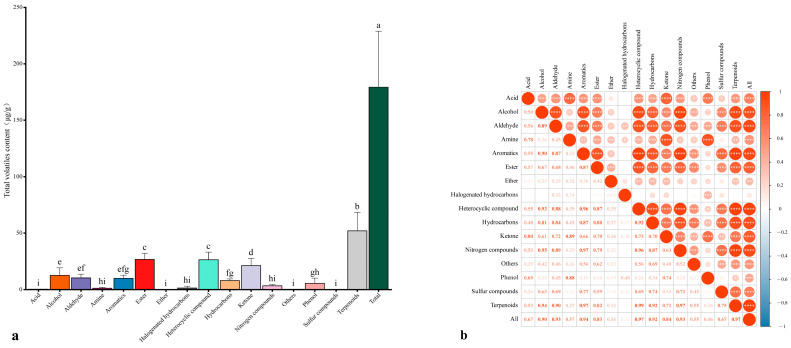
Volatile compound content and correlation analysis of the fruits of 24 apricot cultivars. (**a**) Histogram of the volatile compound contents in the fruits of 24 apricot cultivars. A statistical analysis was performed via a one-way analysis of variance (ANOVA), and different letters indicate significant differences at the 0.05 level. (**b**) Heatmap of the correlation between the content of 16 volatile compounds and the content of total volatile compounds; * represents a significant correlation when *p* < 0.05, ** *p* < 0.01, *** *p* < 0.001, and **** *p* < 0.0001, and color depth represents Pearson’s phase relationship value. Each value is the mean ± standard deviation of three biological replicates.

The aroma characteristics of fruits are not only related to the types of volatile compounds present but also closely related to the content of volatile compounds. We analyzed the metabolism of various volatile compounds in the fruits of different apricot cultivars, and the components and contents widely varied among the cultivars (Supplementary Figure S3). Terpenes were the main volatile compounds in the apricot fruits, with an average total content of 51.93 ± 16.45 µg/g, ranging from 27.23 µg/g (‘ZSHYX’) to 96.03 µg/g (‘JNL’). Esters were the second most dominant aroma compound, with an average total volatile content of 26.73 ± 5.35 µg/g and a range of 39.95 µg/g (‘JNL’) to 19.99 µg/g (ZSHYX’). The heterocyclic compounds ranked third, with an average concentration of 26.44 ± 6.69 µg/g and a range of 15.939 µg/g (‘ZSHYX’) to 43.836 µg/g (‘JNL’). The ketone content was 21.19 ± 6.40 µg/g on average and ranged from 13.11 µg/g (‘BS’) to 36.55 µg/g (‘JNL’). Alcohols were the fifth most abundant volatile compound and had an average total content of 12.61 ± 6.65 µg/g and a range of 4.20 µg/g (‘YXB’) to 28.91 µg/g (‘JNL’) (Figure 2a).

To confirm the contribution of the above volatile compounds to the total volatile compound content, we conducted Pearson’s correlation analysis between the total volatile components and all volatile components. Figure 2b shows that the contents of total volatile compounds were significantly positively correlated with those of terpenoids (r = 0.97), heterocyclic compounds (r = 0.97), aromatic compounds (r = 0.94), aldehydes (r = 0.93), nitrogen compounds (r = 0.93), hydrocarbons (r = 0.92), and alcohols (r = 0.90). Moreover, the total volatile compound content was weakly positively correlated with the halogenated hydrocarbon content (r = 0.13), ether compound content (r = 0.36), and phenolic compound content (r = 0.46). These results indicated that the terpenoids, heterocyclic compounds, aromatics, nitrogen compounds, aldehydes, alcohols, and hydrocarbons were the main volatile compounds in apricot fruits.

### 3.5. Identification of the Relative Odor Activity Values in Apricot Cultivars

The rOAV can be used to analyze the contributions of volatile components to the aroma of apricots. The levels of all volatile components detected in the fruits of 24 apricot cultivars were evaluated. A total of 42 key aroma compounds with rOAVs greater than 1 were found, and most of the compounds had outstanding aroma properties, such as green, fruity, and woody odors (Table 2). The 42 key aroma compounds could be separated into 7 categories, including 9 terpenoids, 6 aldehydes, 7 esters, 8 heterocyclic compounds, 6 alcohols, 2 aromatics, 1 ketone, and 3 phenol compounds. To clarify the fruit flavor characteristics of the different apricot germplasms, the aroma characteristics of the different apricot germplasms were determined according to the total rOAV of the aroma components of the same aroma; the total rOAV represents the total intensity of each aroma type (Supplementary Figure S4). The 24 apricot germplasm resources included 10 main types of fragrances: nutty, citrusy, fatty, waxy, woody, herbal, floral, fruity, green, and sweet. Citrusy, fatty, and green aromas contributed the most to the aroma characteristics of apricot fruits.

We analyzed the rOAV contributions of the compounds, and the (2S,4R)-4-methyl-2-(2-methylprop-1-enyl)oxane, (E)-non-2-enal, (3-methyl-3-sulfanylbutyl) formate, and thiophen-2-ylmethanethiol contents were greater than 1000; these results indicated that these substances greatly contributed to the overall aroma of the apricot fruits. The compounds with 100 < rOAV < 1000 included 4-methoxybenzaldehyde, 2-nonenal, 2-ethoxy-3-methylpyrazine, 1-thiophen-2-ylethanone, and 4-(2,6,6-trimethylcyclohexen-1-yl) butan-2-one and 4-methylphenol.

### 3.6. Principal Component Analysis and Heatmap Analysis

**Figure 3:**
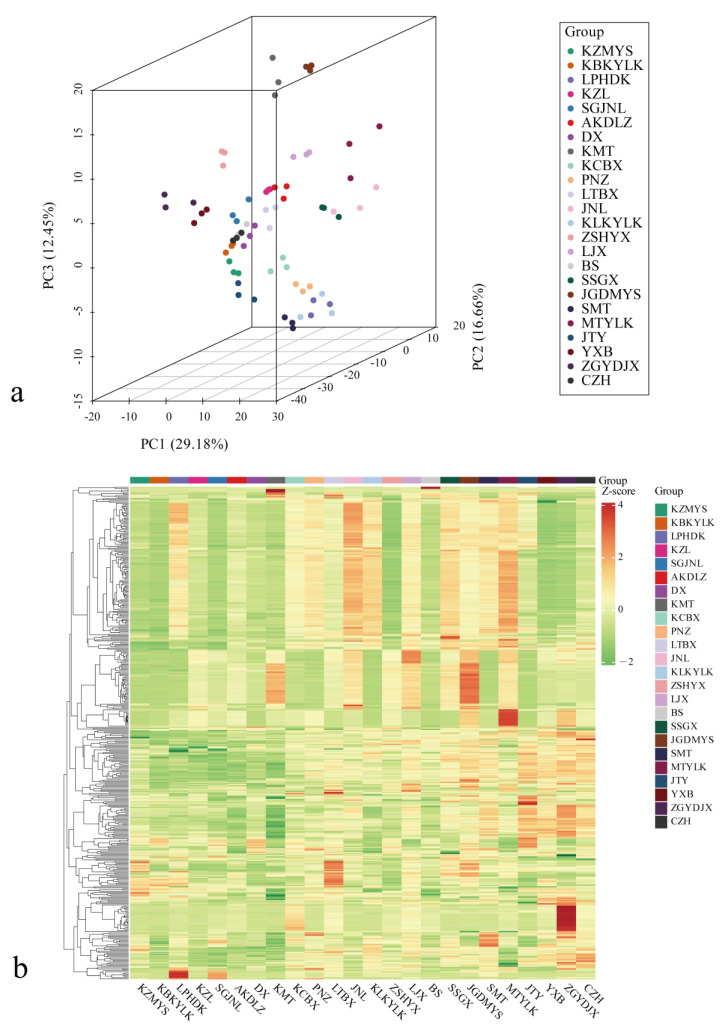
(**a**) Principal component analysis of the volatile compounds in the fruits of 24 apricot cultivars and (**b**) clustering heatmaps of the volatile compounds in the fruits of 24 apricot cultivars. In this figure, the cultivar name is shown in the horizontal direction, the volatile compound information is presented in the vertical direction, the group is under the group column, and the different colors correspond to different values obtained after the standardization treatment of different relative contents (red represents high content, and green represents low content).

To obtain more accurate and intuitive classifications of the volatile components present, PCA was performed for the volatile compounds in the fruits of the 24 apricot cultivars. The PCA diagram clearly reflects the clustering and distribution of different apricot cultivars (Figure 3a), and the principal components explain the changes in the volatile compounds in apricot fruits. Principal component analysis revealed three principal components (PCs), and PC1, PC2, and PC3 accounted for 29.18%, 16.66%, and 12.45% of the total variables, respectively. The cumulative variance contribution rate reached 58.29%, and the distribution of samples in each group was relatively concentrated, indicating good repeatability of samples within groups. Evident separation was found among the 24 tested apricot cultivars. The results indicated that the volatile compounds differed among the cultivars. MTYLK and KMT were distant from those of the other 22 apricot cultivars; these results indicated that the volatile compound contents of the MTYLK and KMT fruits were considerably different from those of the fruits of the other cultivars.

To directly show subtle differences in the contents and components of the mature fruits of the 24 apricot cultivars, a cluster heatmap of 429 volatile aroma compounds was generated (Figure 3b). Lower contents of the volatile aroma compounds correlate to a greener color, whereas higher contents correlate to a redder color; these results reflect differences between the different cultivars and the contents of the volatile compounds.

### 3.7. Correlation Between the Sensory Evaluations and HS–SPME–GC–MS

**Figure 4:**
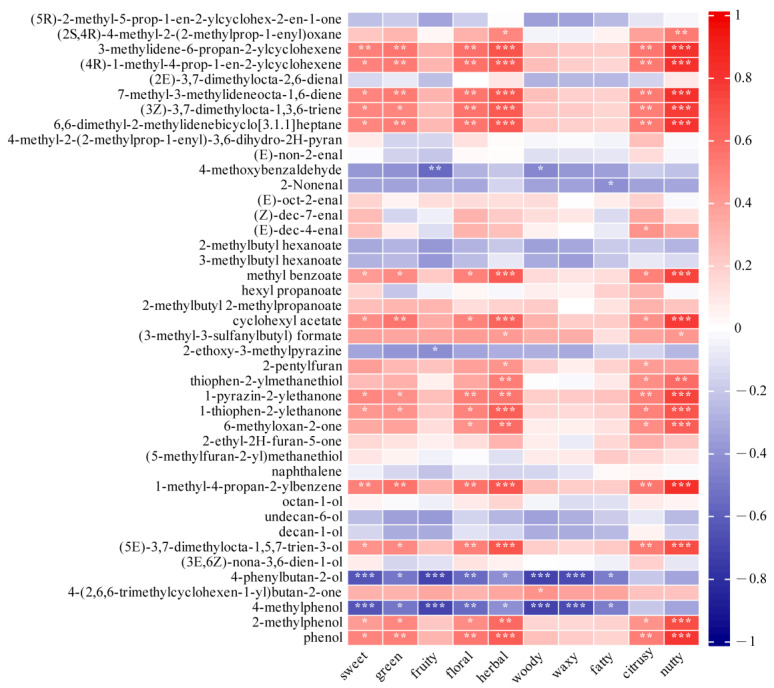
Correlation analysis of the aroma compounds with sensory evaluation results on the basis of Pearson’s rank correlation (* represents a significant difference at *p* < 0.05, ** *p* < 0.01, and *** *p* < 0.001, and color depth represents the Pearson phase relationship value).

Due to their high rOAV based on the GC–MS analysis, forty-two key aroma volatile compounds were selected to be associated with the sensory evaluation results via Pearson’s rank correlation, as shown in Figure 4. Most terpenoids, such as 6,6-dimethyl-2-methylidenebicyclo [3.1.1]heptane, 7-methyl-3-methylideneocta-1,6-diene, and 3-methylidene-6-propan-2-ylcyclohexene, were positively correlated with nutty, herbal, and green aromas. In addition, esters such as thiophen-2-ylmethanethiol, 1-thiophen-2-ylethanone, and 6-methyloxan-2-one were also positively associated with floral, herbal, and nutty compounds; these results were consistent with the HS-SPME-GC–MS results.

## 4. Discussion

Fruit flavor involves aroma and taste; aroma is determined by the numerous volatile substances, and taste is determined by the organic acids and sugars [22]. At present, many reports exist on the use of HS-SPME-GC–MS to analyze volatile components in fruits. For example, Li et al. [23] used HS-SPME-GC–MS to analyze the volatile organic compounds of passion fruit at different maturity stages and identified 148 volatile compounds in passion fruit at the maturity stage; among these compounds, the contribution rate of the ester compound content was the highest. Lan et al. [24] used HS-SPME-GC–MS to detect 172 aroma chemical compositions from 23 kiwifruit varieties, and the identified components could be divided into 9 categories. Sensory assessment is the most reliable technique for converting human perceptions of the aroma, appearance, taste, and aftertaste of food into digital data [25]. In this study, the volatile compounds of 24 apricot cultivars were analyzed via sensory evaluation combined with volatile metabolomic analysis via HS-SPME-GC–MS, and 429 volatile aromatic compounds were detected and could be separated into 16 main categories. Tang et al. [11] detected and identified 15 volatile compounds in apricot fruits. Solís-Solís et al. [12] used simultaneous distillation extraction (SDE), solid-phase extraction (SPE), and liquid–liquid extraction (LLE) methods to detect the volatile components of 8 different varieties of apricot fruits and identified 21, 16, and 8 volatile compounds, respectively. These results were likely caused by the different detection varieties or detection methods and resulted in differences in the amount of compounds detected. For example, Zhao et al. [26]. determined the volatile compounds in the fruits of 4 apricot varieties in Xinjiang using HS-SPME-GC–MS technology and identified a total of 63 volatile compounds; these results likely occurred because of the use of different databases.

The aromas of fruits of different cultivars of the same tree species can differ. In this study, volatile metabolomics combined with the rOAV method was used to analyze the volatile components in the fruits of 24 apricot cultivars. Terpenoids were predominant in the fruits of the apricots and accounted for 17.95% of the total volatile components, followed by 70 esters; the esters accounted for 16.32% of the total volatile components. These percentages were consistent with the results from Zhao et al. and Aubert et al. [27,28]. However, Ayour et al. [29] identified volatile components in the fruits of 10 apricot clones from Morocco, and their results revealed that the dominant components in these fruits were aldehydes, alcohols, and acetic esters. Their results differed from those of this study, and the difference were likely caused by genetic factors or different environmental conditions. Similar findings have been reported for other tree species. For example, Giannetti et al. [30] detected and analyzed the volatile substances of 42 apple fruits in Italy and reported that ester compounds were their main volatile compounds. However, Liu et al. [31] reported that aldehyde volatile compounds were the main volatile substances in ‘Ruixue’ apples. These findings confirmed that the key aroma compounds in fruits of the same tree species and different cultivars greatly differed because of different genetic backgrounds.

The types and contents of volatile compounds may be related to cultivation conditions and climate factors, and substantial differences exist among the different varieties in the same producing region [32]. Wang et al. [33] studied the volatile compositions of the fruits of pear cultivars from different regions and reported considerable differences in the volatile components of these fruits. In Malatya, SPME-GC–MS was used to detect important aroma compounds in the fruits of 15 cultivated apricot varieties, and large differences were observed in the volatile compounds in the fruits of apricot varieties grown under the same conditions; their results were in agreement with those from this study [34]. Thus, under the same cultivation environment, the contents of volatile components in the fruits of 24 apricot cultivars greatly varied and ranged from 112.77 to 317.67 µg/g. Therefore, cultivation conditions and genetic background are likely important factors leading to differences in volatile compounds.

The aroma contribution of a volatile organic compound to fruits depends not only on the volatile species and its content but also on its odor threshold [35]. The rOAV method has been widely used to quantitatively evaluate the odor contribution of aromatic compounds [36,37,38]. In addition, the rOAV method was used to identify the volatile compounds of 24 apricot cultivars. Ninety-three key aroma compounds were identified, and terpenoids, esters, aldehydes, and heterocyclic compounds were the key aroma compounds. In addition, substances with rOAVs above 1000 were screened. Here, (2S,4R)-4-methyl-2-(2-methylprop-1-enyl)oxane, (E)-non-2-enal, (3-methyl-3-sulfanylbutyl) formate, and thiophen-2-ylmethanethiol were identified as key aroma compounds. These results were inconsistent with those from Greger and Schieberle’s study; these differences occurred because the upgraded plant metabolism database used in this study was more abundant and comprehensive [14]. In previous studies, key volatile compounds of mulberry [39], peach [27], and Gannan navel orange [40] were identified via the rOAV method. In our study, we confirmed the practicability and accuracy of this method from the perspective of the key aroma compounds in apricot fruit.

## 5. Conclusions

In summary, sensory evaluation revealed that the apricot fruits had typical fruity, sweet, and green aromas. A total of 429 volatile compounds were detected through qualitative and quantitative analyses of 24 apricot cultivars via the HS-SPME-GC–MS metabolomics method. These results indicated that terpenoids, heterocyclic compounds, aromatics, nitrogen compounds, aldehydes, alcohols, and hydrocarbons were the main volatile compounds in the apricot fruits. Forty-two key volatile compounds were screened according to their aroma activity, and the total volatile compound content in the fruits of 24 apricot cultivars ranged from 112.77 to 317.67 µg/g. The apricot cultivars with the highest and lowest total volatile compound contents were ‘JNL’ (317.67 µg/g) and ‘ZSHYX’ (112.77 µg/g), respectively. Forty-two key aroma compounds contributing to the aroma of apricot fruits were identified via the rOAV method, and (2S,4R)-4-methyl-2-(2-methylprop-1-enyl)oxane, (E)-non-2-enal, (3-methyl-3-sulfanylbutyl) formate, and thiophen-2-ylmethanethiol were determined to be the major active aroma compounds in apricot fruits. These results greatly contribute to the study of volatile metabolites in apricot fruits and reveal apricot germplasm resources with strong aromas; thus, our study provides a theoretical basis for apricot breeding and can aid in the improvement of apricot fruit quality.

## Figures and Tables

**Table 1:** Fruit quality indices of 24 apricot cultivars ^A^.

Number	Cultivar	Harvest Date	Single-Fruit Weight (g)	Soluble Solids (%)	pH	Fruit Firmness (kg/cm^2^)	Fruit Shape Index (%)	Fruit Water Content (%)	Edible Rate (%)
KZMYS	Kezimayisang	11 June	18.52 ± 0.64 ^m^	17.96 ± 0.53 ^efg^	4.33 ± 0.17 ^cdef^	4.73 ± 0.19 ^cd^	0.99 ± 0.00 ^efg^	81.76 ± 0.04 ^efg^	89.60 ± 0.01 ^ijkl^
KBKYLK	Kabakeyulvke	11 June	55.48 ± 2.01 ^c^	10.96 ± 0.54 ^i^	4.13 ± 0.11 ^cdef^	1.16 ± 0.09 ^j^	0.96 ± 0.01 ^fghi^	91.73 ± 0.01 ^a^	93.01 ± 0.01 ^bc^
LPHDK	Luopuhongdaike	11 June	23.17 ± 0.55 ^jkl^	17.34 ± 1.04 ^fg^	4.30 ± 0.12 ^cdef^	5.50 ± 0.23 ^b^	0.94 ± 0.01 ^hij^	85.96 ± 0.01 ^bcd^	90.32 ± 0.01 ^hijk^
KZL	Kezilang	16 June	48.90 ± 1.40 ^ld^	16.46 ± 0.55 ^fgh^	4.38 ± 0.13 ^bcde^	4.44 ± 0.24 ^de^	1.06 ± 0.01 ^cd^	83.33 ± 0.01 ^def^	92.59 ± 0.01 ^bcdef^
SGJNL	Suogejianali	20 June	28.95 ± 1.06 ^hi^	12.42 ± 0.57 ^i^	5.10 ± 0.04 ^a^	5.44 ± 0.20 ^b^	0.98 ± 0.01 ^efgh^	87.33 ± 0.02 ^bc^	92.40 ± 0.01 ^bcdefg^
AKDLZ	Akedalazi	21 June	22.63 ± 0.34 ^klm^	25.34 ± 0.62 ^a^	4.54 ± 0.13 ^abc^	5.58 ± 0.29 ^b^	1.12 ± 0.01 ^b^	73.17 ± 0.03 ^j^	90.95 ± 0.02 ^fghijk^
DX	Danxing	22 June	30.81 ± 0.74 ^h^	21.12 ± 0.55 ^bcd^	3.63 ± 0.18 ^f^	5.38 ± 0.13 ^b^	0.92 ± 0.01 ^ijk^	79.23 ± 0.06 ^gh^	87.65 ± 0.02 ^n^
KMT	Kumaiti	26 June	21.31 ± 0.75 ^lm^	24.80 ± 2.33 ^a^	5.08 ± 0.22 ^ab^	5.58 ± 0.20 ^b^	1.07 ± 0.01 ^c^	76.41 ± 0.03 ^hij^	92.88 ± 0.01 ^bcde^
KCBX	Kuchebaixing	26 June	27.22 ± 0.62 ^hij^	23.08 ± 1.11 ^abc^	4.62 ± 0.18 ^abc^	4.68 ± 0.15 ^cd^	1.12 ± 0.01 ^b^	74.69 ± 0.03 ^j^	91.32 ± 0.01 ^defgh^
PNZ	Pinaizi	30 June	47.45 ± 2.48 ^d^	20.64 ± 0.55 ^cde^	4.40 ± 0.21 ^abcd^	6.44 ± 0.23 ^a^	0.95 ± 0.02 ^ghij^	78.69 ± 0.01 ^gh^	91.22 ± 0.01 ^fghij^
LTBX	Luntaibaixing	1 July	25.24 ± 0.39 ^ijkl^	23.66 ± 2.08 ^abc^	4.74 ± 0.14 ^abc^	5.18 ± 0.14 ^bc^	1.02 ± 0.01 ^de^	81.81 ± 0.02 ^efg^	91.26 ± 0.00 ^efghi^
JNL	Jianali	3 July	41.68 ± 1.46 ^f^	16.10 ± 0.60 ^gh^	4.26 ± 0.08 ^abcd^	3.86 ± 0.10 ^fg^	1.05 ± 0.01 ^cd^	81.80 ± 0.01 ^efg^	90.86 ± 0.01 ^ghijk^
KLKYLK	Kalakeyulvke	4 July	22.67 ± 0.48 ^klm^	25.04 ± 0.75 ^a^	3.71 ± 0.13 ^def^	4.52 ± 0.06 ^de^	1.00 ± 0.03 ^ef^	73.89 ± 0.02 ^j^	89.38 ± 0.01 ^klm^
ZSHYX	Zaoshuheiyexing	5 July	46.30 ± 1.09 ^de^	17.68 ± 0.73 ^efg^	4.46 ± 0.13 ^abc^	4.04 ± 0.33 ^efg^	1.07 ± 0.01 ^c^	78.40 ± 0.01 ^ghi^	89.56 ± 0.00 ^jkl^
LJX	Lajiaoxing	6 July	42.69 ± 0.99 ^ef^	16.46 ± 1.09 ^fgh^	4.57 ± 0.07 ^abc^	4.30 ± 0.13 ^def^	1.17 ± 0.03 ^a^	78.48 ± 0.01 ^ghi^	91.89 ± 0.01 ^cdefgh^
BS	Beishan	6 July	37.68 ± 1.03 ^g^	19.66 ± 0.72 ^def^	4.70 ± 0.12 ^abc^	4.04 ± 0.33 ^efg^	0.99 ± 0.02 ^efgh^	84.52 ± 0.01 ^bcde^	87.96 ± 0.01 ^mn^
SSGX	Shushangganxing	8 July	22.99 ± 0.75 ^jkl^	25.54 ± 1.37 ^a^	4.59 ± 0.05 ^abc^	3.08 ± 0.11 ^hi^	0.97 ± 0.01 ^fgh^	75.24 ± 0.02 ^ij^	90.64 ± 0.01 ^hijk^
JGDMYS	Jiagedamayisang	9 July	42.64 ± 1.14 ^ef^	24.20 ± 0.63 ^ab^	4.56 ± 0.11 ^abc^	2.76 ± 0.11 ^i^	1.13 ± 0.01 ^ab^	78.67 ± 0.05 ^gh^	92.95 ± 0.01 ^bcd^
SMT	Saimaiti	11 July	26.03 ± 0.89 ^ijk^	17.58 ± 1.10 ^efg^	4.14 ± 0.11 ^cdef^	4.62 ± 0.21 ^d^	1.05 ± 0.01 ^cd^	79.99 ± 0.01 ^fg^	88.08 ± 0.01 ^lmn^
MTYLK	Mantouyulvke	9 July	30.90 ± 1.25 ^h^	26.04 ± 1.43 ^a^	4.54 ± 0.06 ^abc^	3.86 ± 0.13 ^fg^	0.98 ± 0.01 ^efgh^	87.80 ± 0.00 ^b^	90.72 ± 0.03 ^ghijk^
JTY	Jintaiyang	20 June	100.91 ± 2.56 ^a^	16.32 ± 0.87 ^fgh^	4.04 ± 0.46 ^cdef^	5.58 ± 0.14 ^b^	0.99 ± 0.01 ^efgh^	84.05 ± 0.01 ^cde^	89.61 ± 0.00 ^ijkl^
YXB	Yinxiangbai	20 June	58.61 ± 2.22 ^c^	16.84 ± 0.72 ^fgh^	3.68 ± 0.48 ^ef^	5.28 ± 0.16 ^b^	0.89 ± 0.01 ^k^	84.25 ± 0.01 ^cde^	96.02 ± 0.01 ^a^
ZGYDJX	Zhanggongyuandajiexing	3 July	70.33 ± 2.55 ^b^	13.72 ± 0.40 ^hi^	4.11 ± 0.46 ^cdef^	3.76 ± 0.18 ^fg^	0.91 ± 0.02 ^jk^	80.37 ± 0.01 ^fg^	91.43 ± 0.01 ^cdefgh^
CZH	Chuanzhihong	4 July	42.85 ± 2.32 ^ef^	13.72 ± 0.57 ^hi^	4.03 ± 0.15 ^cdef^	3.58 ± 0.11 ^gh^	1.06 ± 0.01 ^cd^	85.52 ± 0.01 ^bcd^	93.68 ± 0.01 ^b^

^A^ Data are expressed as the mean ± standard deviation of 3 biological replicates (*p* < 0.05). Different lowercase letters indicate significant differences at the 0.05 level.

**Table 2:** Key volatile compounds with rOAVs greater than 1 in 24 apricot cultivars ^a,b^.

Volatile Compounds	Odor Description	Threshold µg/g	KZMYS	KBKYLK	LPHDK	KZL	SGJNL	AKDLZ	DX	KMT	KCBX	PNZ	LTBX	JNL	KLKYLK	ZSHYX	LJX	BS	SSGX	JGDMYS	SMT	MTYLK	JTY	YXB	ZGYDJX	CZH
Terpenoids																										
(5R)-2-methyl-5-prop-1-en-2-ylcyclohex-2-en-1-one	sweet, spearmint, herbal, minty	0.085	22.41	21.14	20.91	21.45	18.29	20.40	19.83	16.84	19.58	23.03	17.97	25.48	17.91	17.21	20.89	18.66	20.11	17.83	20.50	18.35	24.49	21.77	20.78	20.47
(2S,4R)-4-methyl-2-(2-methylprop-1-enyl)oxane	rose, cortex, green, floral, geranium, powdery, metallic	0.0002	3771.60	3403.88	5905.12	4688.88	2823.62	3901.08	3359.75	2507.70	3927.37	4460.41	3160.84	6420.09	3878.22	2045.42	4027.48	2773.81	4043.41	3211.68	3883.88	3873.36	4537.55	2449.56	2690.79	3003.40
3-methylidene-6-propan-2-ylcyclohexene	terpenic, herbal	0.036	9.62	7.19	53.43	27.03	8.22	37.03	18.86	15.82	41.74	49.30	24.07	76.22	50.00	1.28	40.42	12.43	50.89	31.35	40.02	62.15	20.44	1.58	2.96	16.76
(4R)-1-methyl-4-prop-1-en-2-ylcyclohexene	citrus	0.034	11.32	8.38	49.24	25.27	8.80	34.70	18.86	16.45	42.84	49.20	26.93	79.08	55.79	3.74	43.55	14.63	58.70	35.95	47.45	68.73	21.65	4.53	7.22	20.45
(2E)-3,7-dimethylocta-2,6-dienal	citrus, lemon	0.028	1.54	1.07	1.25	1.16	1.09	1.23	1.22	1.10	1.18	1.24	1.11	1.57	1.26	1.03	1.32	1.15	1.38	1.20	1.22	1.08	1.46	1.34	1.26	1.29
7-methyl-3-methylideneocta-1,6-diene	musty, balsamic, spice	0.015	10.17	9.56	36.02	18.03	9.41	23.72	14.28	14.23	29.03	34.11	19.75	52.33	33.67	5.89	31.35	14.29	40.51	26.18	31.43	48.65	18.74	6.84	8.53	16.31
(3Z)-3,7-dimethylocta-1,3,6-triene	warm, floral, herbal, flowery, sweet	0.034	9.15	6.25	53.63	22.55	7.00	26.45	16.60	12.04	32.41	45.89	18.44	80.70	48.71	1.51	37.53	8.79	49.58	27.66	37.81	64.65	17.21	1.70	2.33	12.10
6,6-dimethyl-2-methylidenebicyclo[3.1.1]heptane	dry, woody, resinous, pine, hay, green	0.14	5.51	4.09	30.18	13.65	4.65	18.25	9.62	8.59	21.15	25.00	13.54	42.92	26.61	1.02	20.84	6.92	29.93	17.69	23.11	36.52	12.88	1.08	2.41	9.41
4-methyl-2-(2-methylprop-1-enyl)-3,6-dihydro-2H-pyran	green, weedy, cortex, herbal, diphenyl, narcissus, celery	0.08	7.95	7.54	6.52	7.46	5.50	4.99	6.00	3.28	5.16	5.83	5.30	6.84	5.22	4.60	6.02	3.17	5.54	4.70	5.19	4.29	5.16	5.38	5.23	5.32
Aldehyde																										
(E)-non-2-enal	fatty, green, cucumber, aldehydic, citrus	0.00008	26,867.54	26,660.46	24,441.08	27,909.35	21,260.59	21,089.75	22,704.10	14,688.58	20,704.07	23,124.84	20,907.21	26,470.78	18,856.92	17,103.23	21,362.19	15,076.52	20,721.52	18,117.62	21,222.03	20,796.90	23,529.57	22,915.28	22,765.57	22,388.08
4-methoxybenzaldehyde	sweet, powdery, mimosa, floral, hawthorn, balsamic	0.0002	687.29	692.46	642.06	738.19	595.01	614.24	630.03	510.19	554.14	579.79	498.00	694.20	515.63	467.00	510.25	514.58	575.76	541.59	651.82	540.45	739.52	686.05	619.79	600.92
2-Nonenal	fatty, green, waxy, cucumber, melon	0.0001	509.62	492.87	462.81	489.54	410.19	420.18	440.02	283.47	410.50	425.58	467.63	480.70	348.99	361.49	499.42	347.51	369.76	368.05	442.95	416.72	602.18	584.35	525.56	391.75
(E)-oct-2-enal	fresh, cucumber, fatty, green, herbal, banana, waxy, leafy	0.003	21.47	5.61	6.69	4.62	4.25	6.22	3.24	3.30	5.04	5.27	16.13	5.54	2.95	2.95	4.42	3.99	5.31	7.66	3.18	5.30	3.27	5.01	2.95	2.92
(Z)-dec-7-enal	citrus, aldehydic, cucumber	0.0022	27.24	20.36	24.17	19.88	19.35	13.92	9.89	6.42	13.69	18.90	19.79	16.57	17.05	8.87	19.63	8.76	19.47	13.78	25.11	12.81	8.86	22.16	12.18	13.07
(E)-dec-4-enal	fresh, aldehydic, citrus, orange, mandarin, tangerine, green, fatty	0.025	4.50	4.46	4.41	4.34	3.14	3.67	3.80	2.81	4.18	5.43	3.42	4.98	5.13	2.82	3.02	3.12	4.17	3.13	5.74	4.40	2.63	3.13	3.26	2.83
Ester																										
2-methylbutyl hexanoate	ethereal	0.032	10.65	10.36	9.44	11.20	9.04	9.18	9.54	7.19	9.21	9.37	7.92	10.93	7.46	7.42	8.86	7.89	7.60	7.45	8.84	7.30	10.91	9.73	10.27	8.64
3-methylbutyl hexanoate	fruity, banana, apple, pineapple, green	0.32	1.97	2.02	1.81	2.07	1.60	1.69	1.74	1.39	1.66	1.83	1.69	2.04	1.47	1.44	1.73	1.52	1.65	1.70	1.71	1.60	2.08	1.87	1.92	1.63
methyl benzoate	phenol, wintergreen, almond, floral, canga	0.00052	529.56	321.85	1861.74	973.59	297.90	937.73	575.36	427.16	1102.52	1201.88	783.10	2468.27	1172.07	77.88	1286.40	396.75	1335.26	1099.57	1257.31	1187.62	962.02	99.98	183.67	497.97
hexyl propanoate	pear, green, fruity, musty, rotten	0.008	13.86	16.24	14.64	8.99	11.26	10.30	8.92	8.38	7.26	11.07	14.23	11.38	8.18	5.94	9.67	9.34	8.98	10.26	7.85	7.25	5.65	11.80	7.94	9.13
2-methylbutyl 2-methylpropanoate	fruity, ethereal, tropical, banana	0.014	5.42	5.27	5.10	4.86	3.80	5.71	4.55	4.08	6.22	4.19	9.59	5.06	4.96	3.64	5.17	3.55	4.84	4.11	4.65	4.32	4.54	4.15	4.24	4.50
cyclohexyl acetate	fruity, sweet, musty, ethereal	0.0016	57.11	60.73	76.80	73.71	55.86	83.92	72.61	66.38	86.54	94.66	70.69	103.19	85.92	54.59	90.51	70.00	92.82	88.92	94.89	127.54	72.12	61.01	74.73	75.50
(3-methyl-3-sulfanylbutyl) formate	sulfur, catty, caramel, onion, roasted coffee, roasted meat, tropical	0.000002	23,748.42	22,618.76	61,174.24	59,536.11	22,496.22	82,847.39	41,518.40	44,328.44	80,447.02	116,085.97	46,860.80	159,809.00	105,693.96	159,809.00	87,642.72	59,317.63	113,129.02	69,779.02	67,231.62	129,673.12	44,795.11	27,531.45	30,570.70	38,475.38
Heterocyclic compound																										
2-ethoxy-3-methylpyrazine	hazelnut, roasted, almond, pineapple, earthy	0.0008	377.56	376.63	293.38	369.85	318.33	329.39	318.45	287.05	298.04	338.20	317.55	404.54	285.32	302.12	324.49	326.91	317.54	337.78	323.54	274.42	364.35	365.76	366.42	330.26
2-pentylfuran	fruity, green, earthy, beany, vegetable, metallic	0.006	94.33	37.64	61.23	42.78	39.38	54.02	36.43	39.58	42.79	58.19	78.20	75.74	41.99	29.12	54.94	48.83	64.12	53.31	51.09	52.08	42.36	39.37	35.26	47.38
thiophen-2-ylmethanethiol	roasted, coffee, fishy	0.00004	21,883.49	22,186.77	34,310.19	26,980.58	18,304.39	22,783.75	20,901.61	16,405.07	22,218.44	25,532.74	22,454.56	38,795.11	22,196.90	12,530.77	25,316.13	16,206.21	25,363.86	22,637.74	24,791.75	22,827.29	26,362.86	14,593.03	16,272.72	17,936.69
1-pyrazin-2-ylethanone	popcorn, nutty, corn, chip, bread, crust, chocolate, hazelnut, coffee	0.01	3.71	4.57	10.50	5.15	4.50	6.56	5.18	5.91	7.12	8.52	4.75	14.93	9.51	2.62	7.22	6.01	9.40	5.67	7.39	12.42	4.49	2.91	2.72	7.44
1-thiophen-2-ylethanone	sulfur, nutty, hazelnut, walnut	0.001	245.17	173.41	920.68	392.43	150.61	395.49	243.01	181.06	467.11	522.41	308.86	976.43	474.25	55.61	441.52	142.74	496.00	367.68	426.20	517.22	320.80	64.52	84.82	182.63
6-methyloxan-2-one	creamy, fruity, coconut	0.02683	6.47	5.49	16.03	8.38	4.55	7.34	5.90	4.90	8.73	10.16	6.98	18.31	9.72	2.91	8.73	4.65	10.01	7.80	9.14	10.46	8.60	3.44	3.85	5.44
2-ethyl-2H-furan-5-one	spice	0.0097	53.56	40.72	45.98	38.12	32.87	41.00	31.05	38.06	36.43	39.14	49.19	47.18	31.11	29.76	38.18	35.47	40.50	45.29	33.08	37.60	38.60	35.95	32.77	29.17
(5-methylfuran-2-yl)methanethiol	sulfur, roasted, coffee	0.00005	976.56	1491.94	974.18	1439.70	844.42	1074.18	1447.34	976.59	1004.76	1223.74	1081.20	1198.54	1149.23	850.56	1100.24	1081.61	1047.08	906.11	1016.13	1233.14	882.90	1080.02	1224.78	1187.38
Aromatics																										
naphthalene	pungent, dry, tarry	0.05	15.74	18.40	16.54	17.98	16.82	17.64	18.27	14.30	15.70	19.33	13.98	18.95	15.05	14.61	15.99	17.51	15.51	15.47	18.18	13.17	16.68	16.75	16.86	19.99
1-methyl-4-propan-2-ylbenzene	woody, citrus	0.0114	26.03	21.56	118.31	64.07	22.64	87.59	49.85	42.56	95.98	110.34	55.28	168.43	113.80	4.24	94.34	38.21	118.88	74.00	96.05	150.57	53.31	4.80	10.94	48.40
Alcohol																										
octan-1-ol	intense citrus, rose	0.022	3.47	2.38	2.46	2.58	2.23	1.94	2.10	1.91	2.21	2.33	3.33	3.08	1.98	1.96	2.43	2.01	2.79	2.36	2.35	2.29	2.74	2.64	2.27	2.14
undecan-6-ol	-	0.0086	8.93	6.31	6.16	6.36	5.42	5.83	5.59	5.48	4.82	4.85	5.08	6.95	6.00	4.94	5.54	5.50	5.42	5.23	5.22	4.22	6.05	5.74	5.94	6.05
decan-1-ol	fatty, waxy, floral, orange, sweet, watery	0.023	5.65	4.37	4.26	4.41	3.74	3.72	3.49	3.14	3.46	4.15	4.07	4.98	4.17	3.73	3.98	3.74	3.84	3.50	3.92	2.91	4.34	3.90	4.03	4.13
(5E)-3,7-dimethylocta-1,5,7-trien-3-ol	sweet, tropical, ocimene, fennel, ginger, myrcene	0.11	32.22	23.34	130.65	55.31	20.59	56.71	32.84	25.34	74.59	89.28	48.69	140.93	63.98	4.60	66.12	19.36	69.80	63.65	61.81	74.05	46.54	5.19	7.76	24.20
(3E,6Z)-nona-3,6-dien-1-ol	fatty, green, cucumber, green pepper, fruity, watermelon	0.003	36.73	26.89	26.92	28.91	19.57	19.71	20.80	12.75	19.70	24.66	23.55	29.13	20.63	17.40	22.78	12.12	15.88	17.26	20.39	16.86	18.25	22.74	22.84	17.39
4-phenylbutan-2-ol	floral, peony, foliage, sweet, mimosa, heliotrope	0.0043	7.97	10.61	7.37	8.55	6.21	6.31	7.06	6.87	5.95	6.23	4.94	7.18	5.37	5.14	6.02	5.21	6.50	5.08	8.29	5.16	11.32	6.26	7.24	5.23
Ketone																										
4-(2,6,6-trimethylcyclohexen-1-yl)butan-2-one	earthy, woody, mahogany, orris, dry, amber	0.0036	19.15	74.11	270.01	570.78	257.37	418.26	24.81	246.32	262.53	419.65	91.23	235.73	11.93	296.37	149.82	16.21	40.86	569.51	93.80	1241.93	3.65	240.95	687.99	267.79
Phenol																										
4-methylphenol	phenol, narcissus, animalic, mimosa	0.00024	234.68	170.26	1186.66	532.67	160.03	557.54	316.89	211.83	686.42	898.13	420.03	1574.38	935.60	53.81	783.76	223.10	983.15	545.64	822.98	1162.24	364.63	78.72	122.28	303.18
2-methylphenol	phenol	0.0039	10.86	11.38	19.88	16.08	10.85	17.23	14.70	12.98	15.87	17.71	13.45	24.47	16.71	10.08	15.51	12.41	17.62	13.61	17.64	20.59	14.97	11.64	13.50	14.56
phenol	phenol, medicinal	0.03	4.32	4.22	19.19	9.04	4.04	13.37	6.82	7.76	15.01	17.53	9.00	25.05	19.49	2.81	14.30	7.46	18.24	11.15	14.95	23.62	7.86	3.90	3.40	9.20

^a^ All odor thresholds were obtained from Tan et al. [21]. ^b^ The aroma of the material is described at http://www.thegoodscentscompany.com

## Data Availability

The original contributions presented in the study are included in the article and Supplementary Materials, further inquiries can be directed to the corresponding author.

## Supplementary Materials

The following supporting information can be downloaded at https://www.mdpi.com/article/10.3390/foods13233912/s1: Supplementary Figure S1. Pictures of the mature fruit of the 24 apricot cultivars; Supplementary Figure S2. Representative total ion chromatogram of a quality control sample (a) and total ion flow diagram of the mixed QC sample (TIC, the sum of the strengths of all ions in the mass spectrometer at each time point) (b)—the horizontal coordinate is the retention time of metabolite detection (RT), and the vertical coordinate is the ion flow intensity of ion detection (CPS); Supplementary Table S1. Relative contents of the identified volatile compounds in the fruits of 24 apricot cultivars; Supplementary Figure S3. Column chart of the volatile compound contents in 24 apricot varieties. A, B, C, D, E, F, G, H, I, J, K, L, M, N, O, and P represent the terpenoids, esters, heterocyclic compounds, ketones, alcohols, aldehydes, aromatics, hydrocarbons, phenols, nitrogen compounds, halogenated hydrocarbons, amines, acids, sulfur compounds, ether, and other compound contents, respectively. Statistical analysis was performed via a one-way analysis of variance (ANOVA), and different letters indicate significant differences at the 0.05 level; and Supplementary Figure S4. Histogram of the aroma characteristics of fruits of different apricot germplasm resources.
