# Supplementary material for: Headspace Solid-Phase Microextraction and Gas Chromatography–Mass Spectrometry Combined with Sensory Evaluation for the Analysis of Volatile Aromatic Compounds in Apricot (Prunus armeniaca L.) Germplasm Resources Cultivated in Xinjiang, China
Source: Foods. 2024 Dec 3;13(23):3912. doi: 10.3390/foods13233912 (PMC11640270; doi:10.3390/foods13233912)
Supplement: Supplementary file 1 [file foods-13-03912-s001.zip › Supplementary Figure S2.pdf]

a

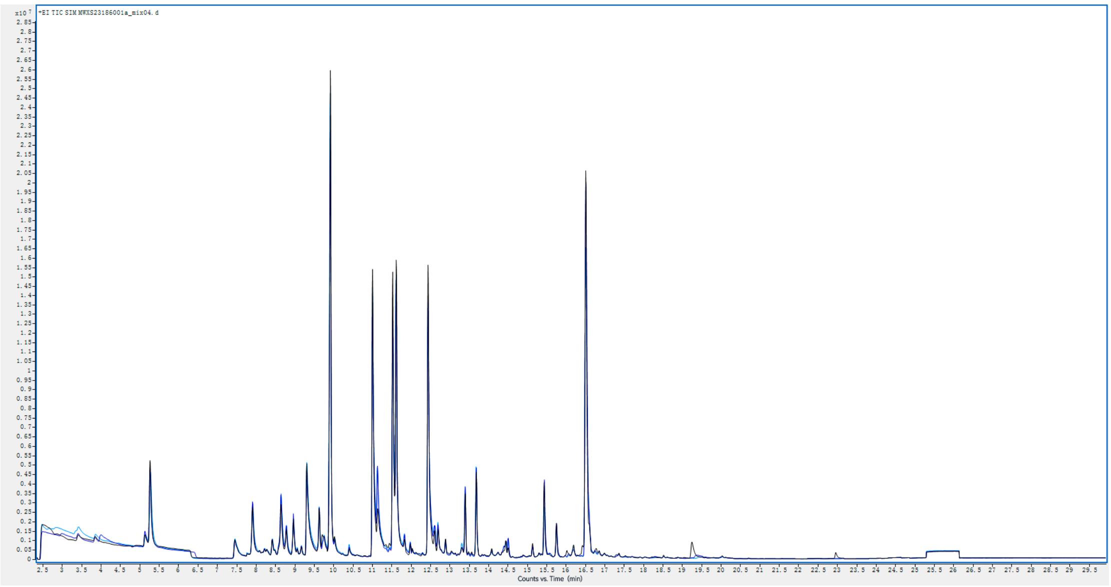

b

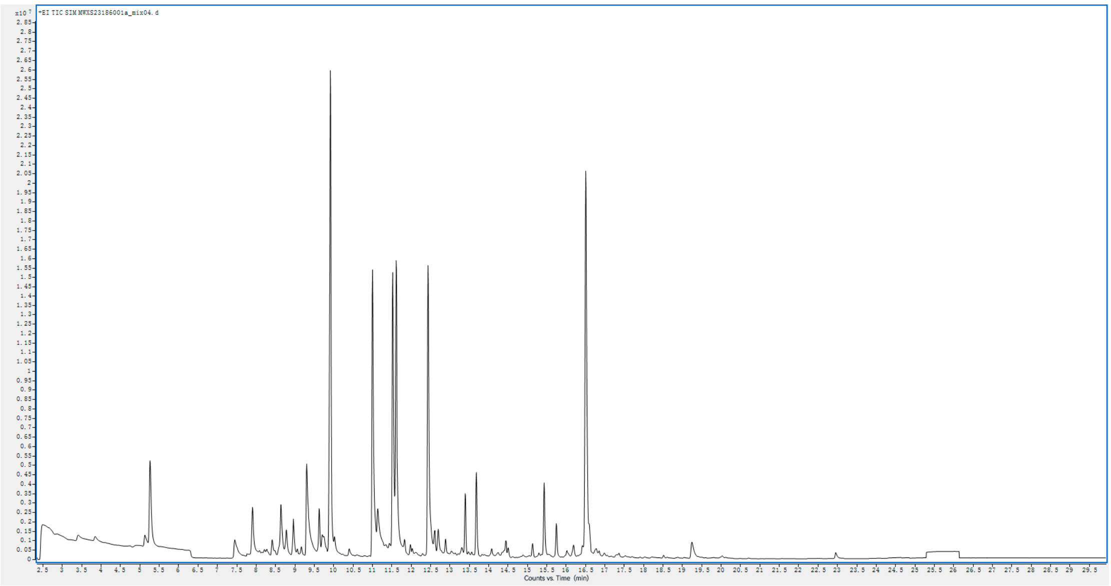

Supplementary Figure S2. Representative total ion chromatogram of a quality control sample (a). The total ion flow diagram of the mixed QC sample (TIC, the sum of the strengths of all ions in the mass spectrometer at each time point) (b); the horizontal coordinate is the retention time of metabolite detection (RT), and the vertical coordinate is the ion flow intensity of ion detection (CPS).
