# Supplementary material for: Headspace Solid-Phase Microextraction and Gas Chromatography–Mass Spectrometry Combined with Sensory Evaluation for the Analysis of Volatile Aromatic Compounds in Apricot (Prunus armeniaca L.) Germplasm Resources Cultivated in Xinjiang, China
Source: Foods. 2024 Dec 3;13(23):3912. doi: 10.3390/foods13233912 (PMC11640270; doi:10.3390/foods13233912)
Supplement: Supplementary file 1 [file foods-13-03912-s001.zip › Supplementary Figure S3.pptx]

## Slide 1
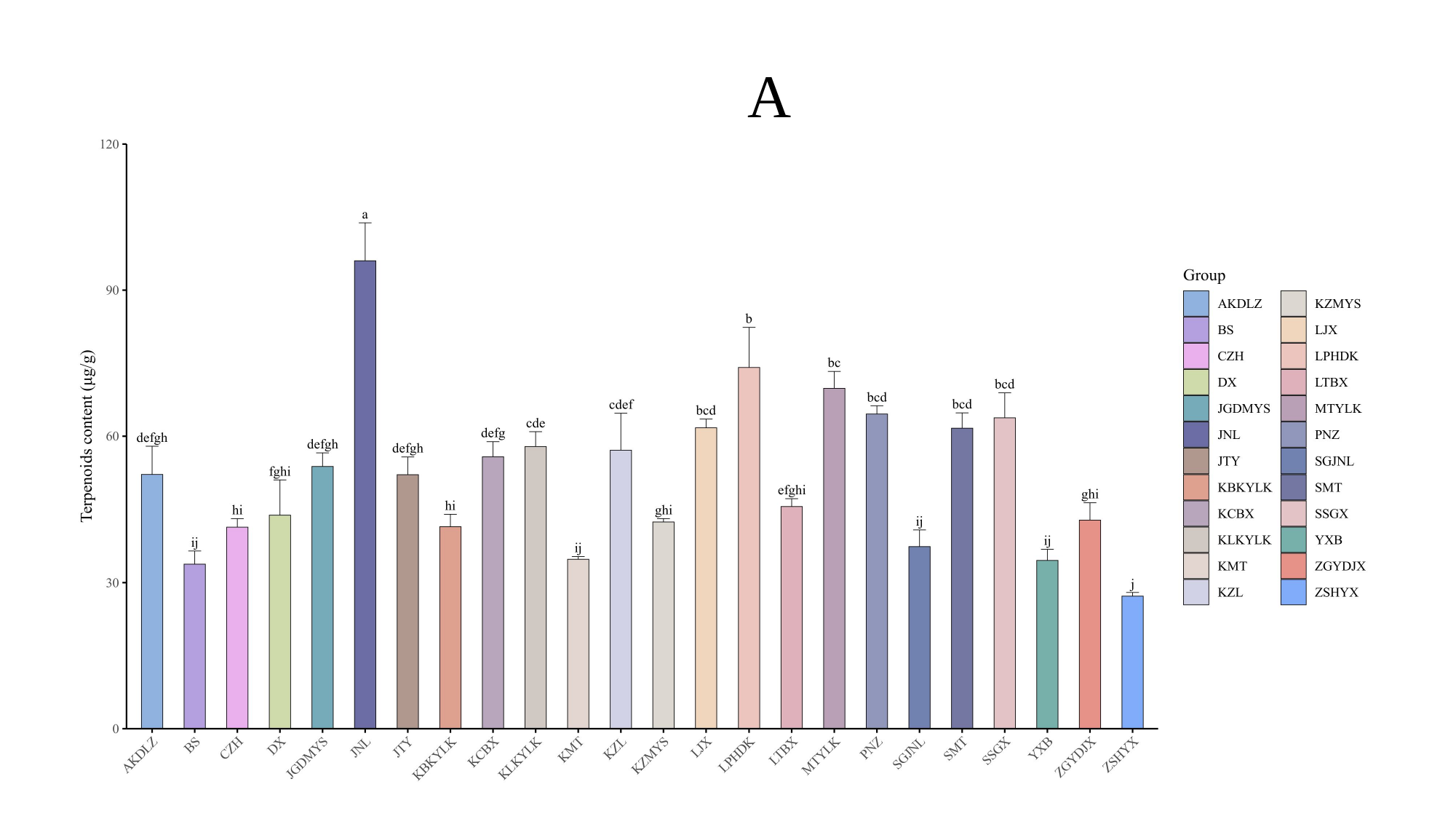

# A

## Slide 2
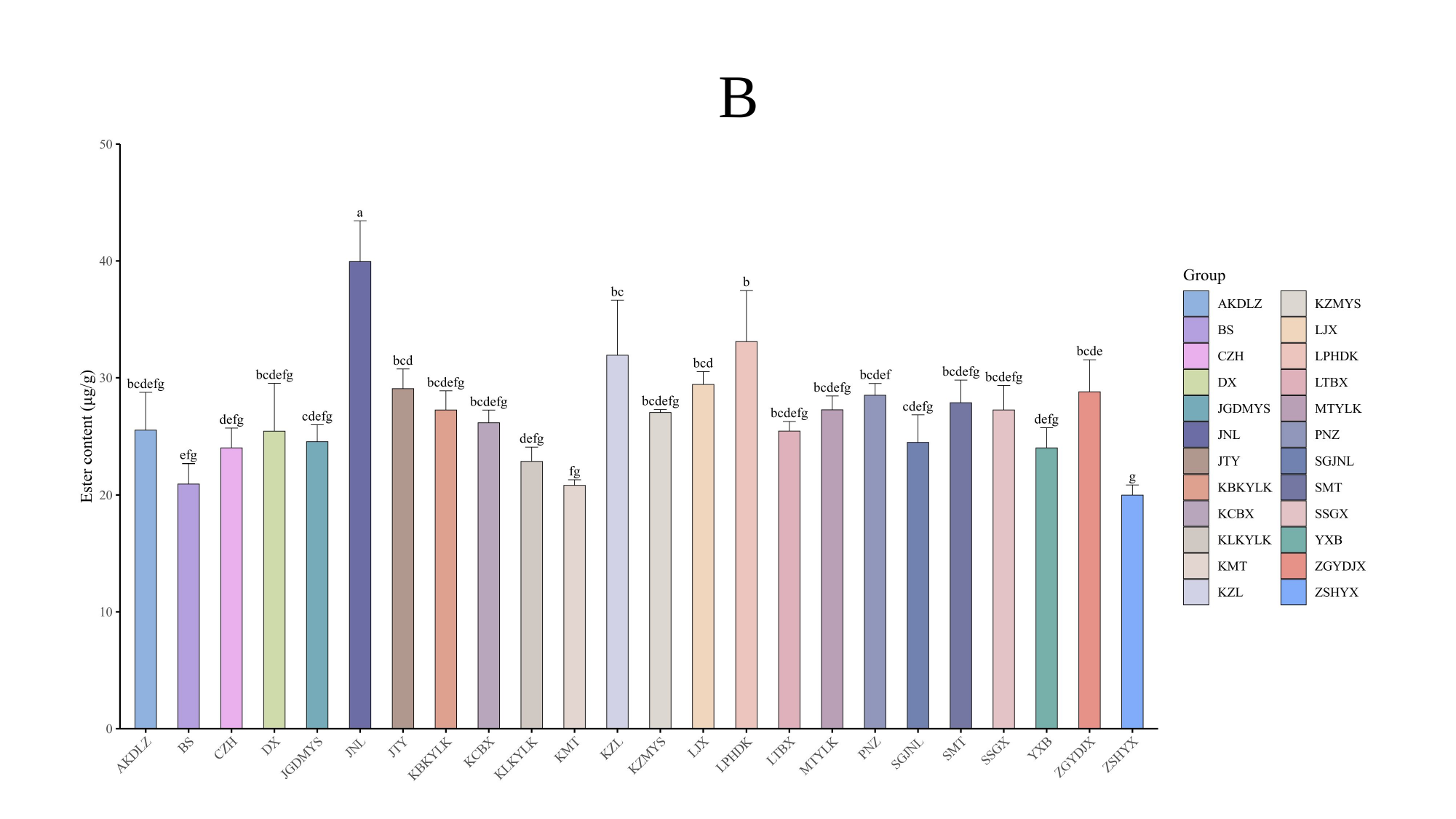

# B

## Slide 3
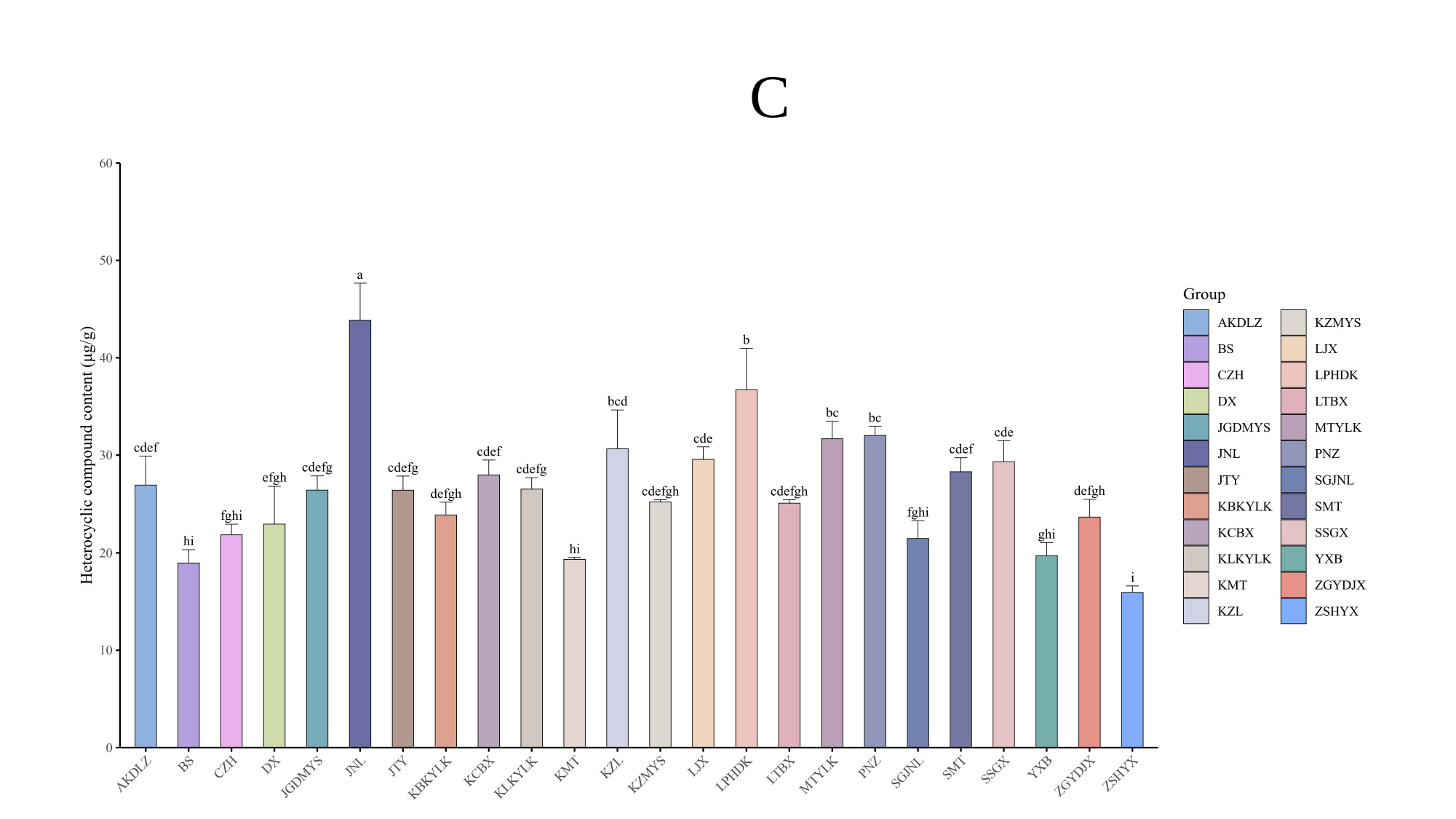

# C

## Slide 4
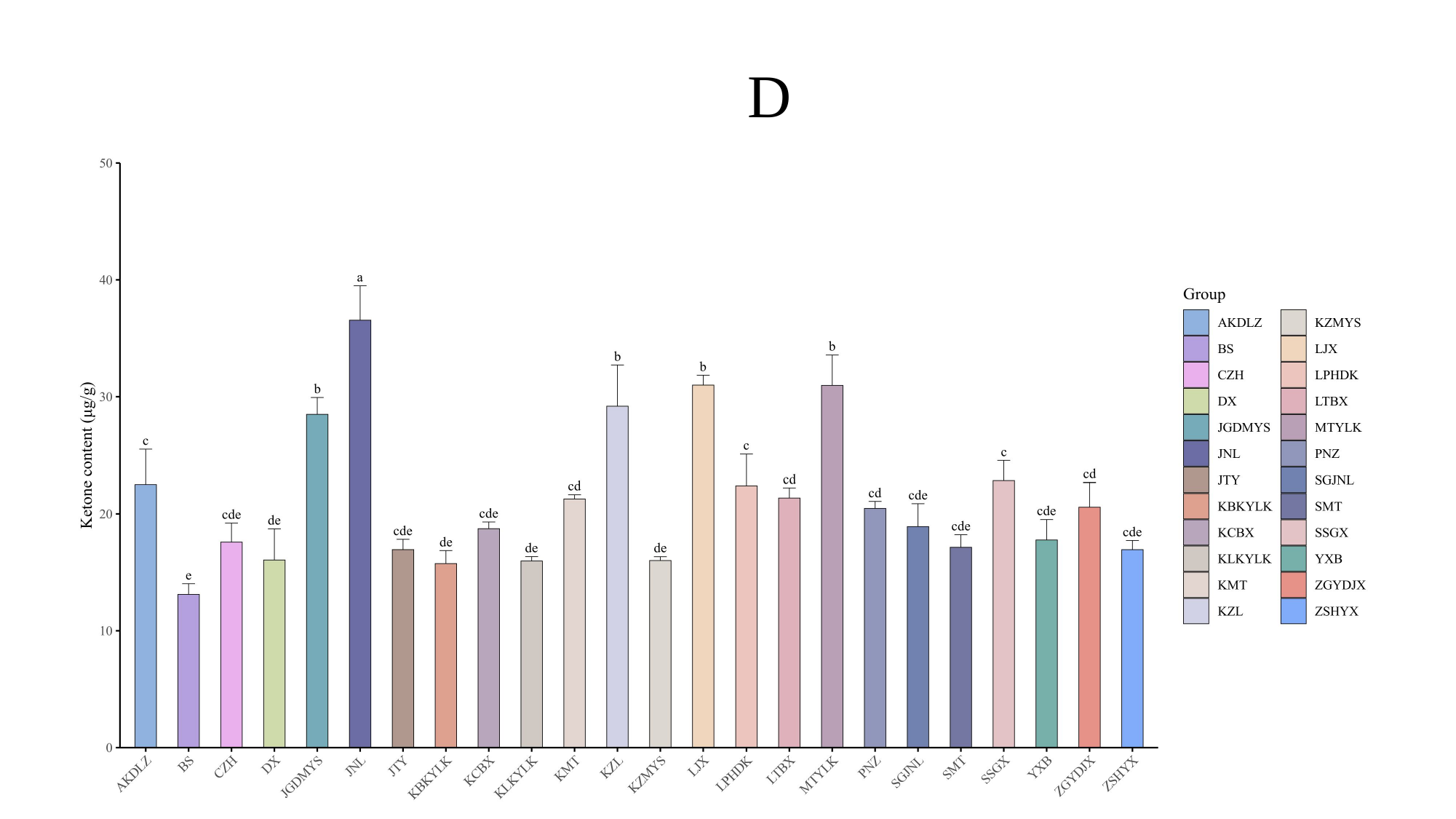

# D

## Slide 5
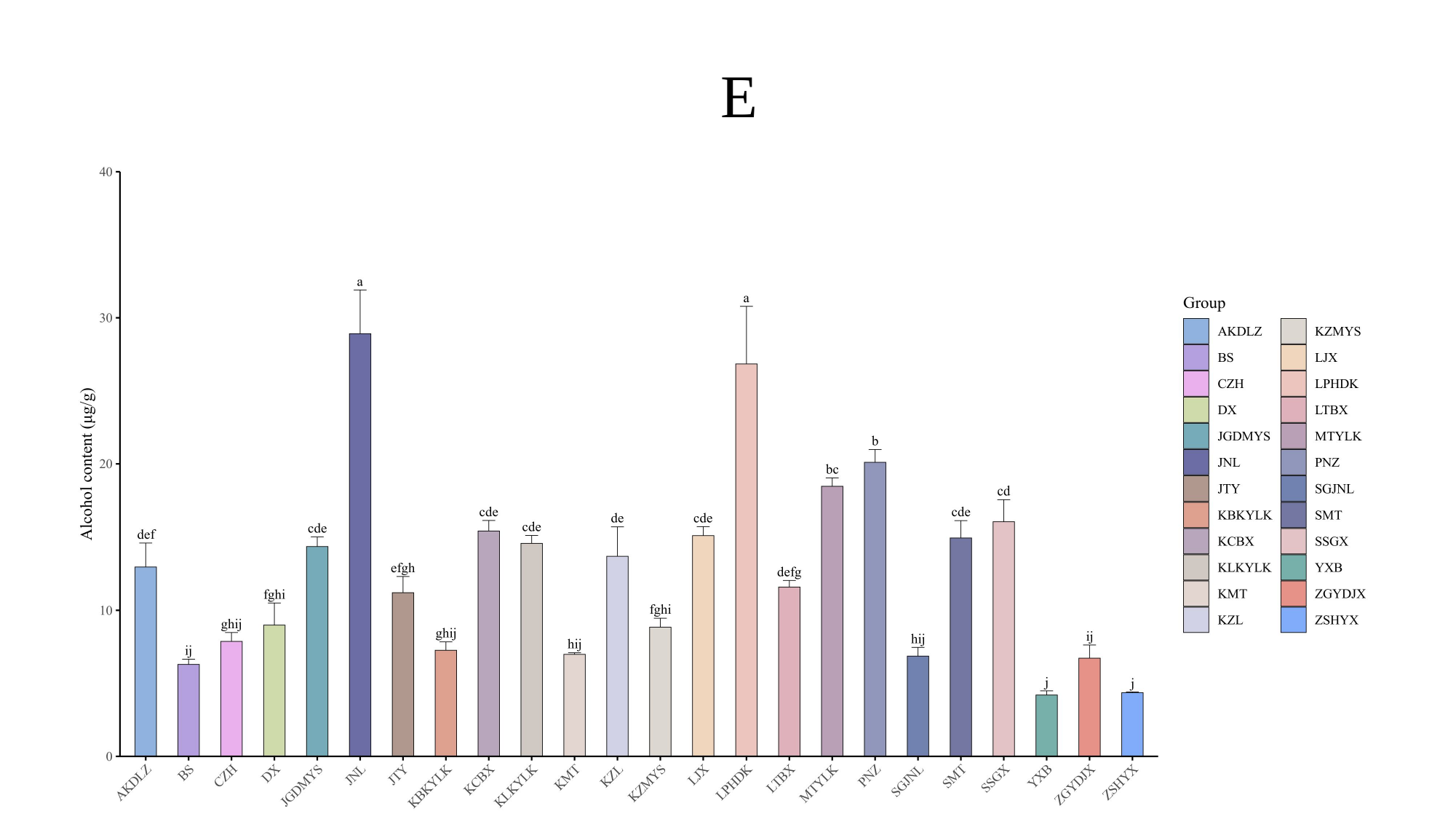

# E

## Slide 6
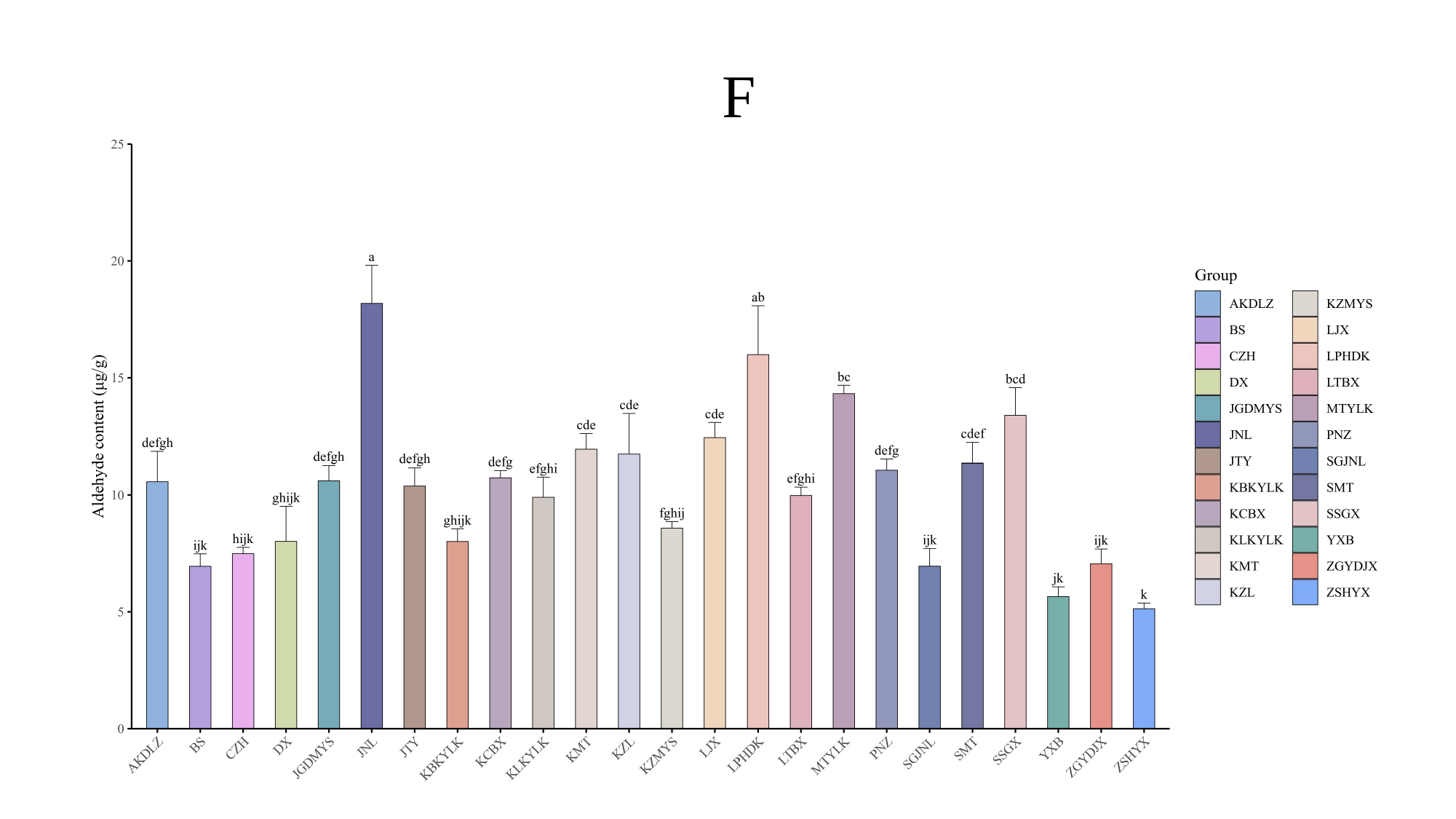

# F

## Slide 7
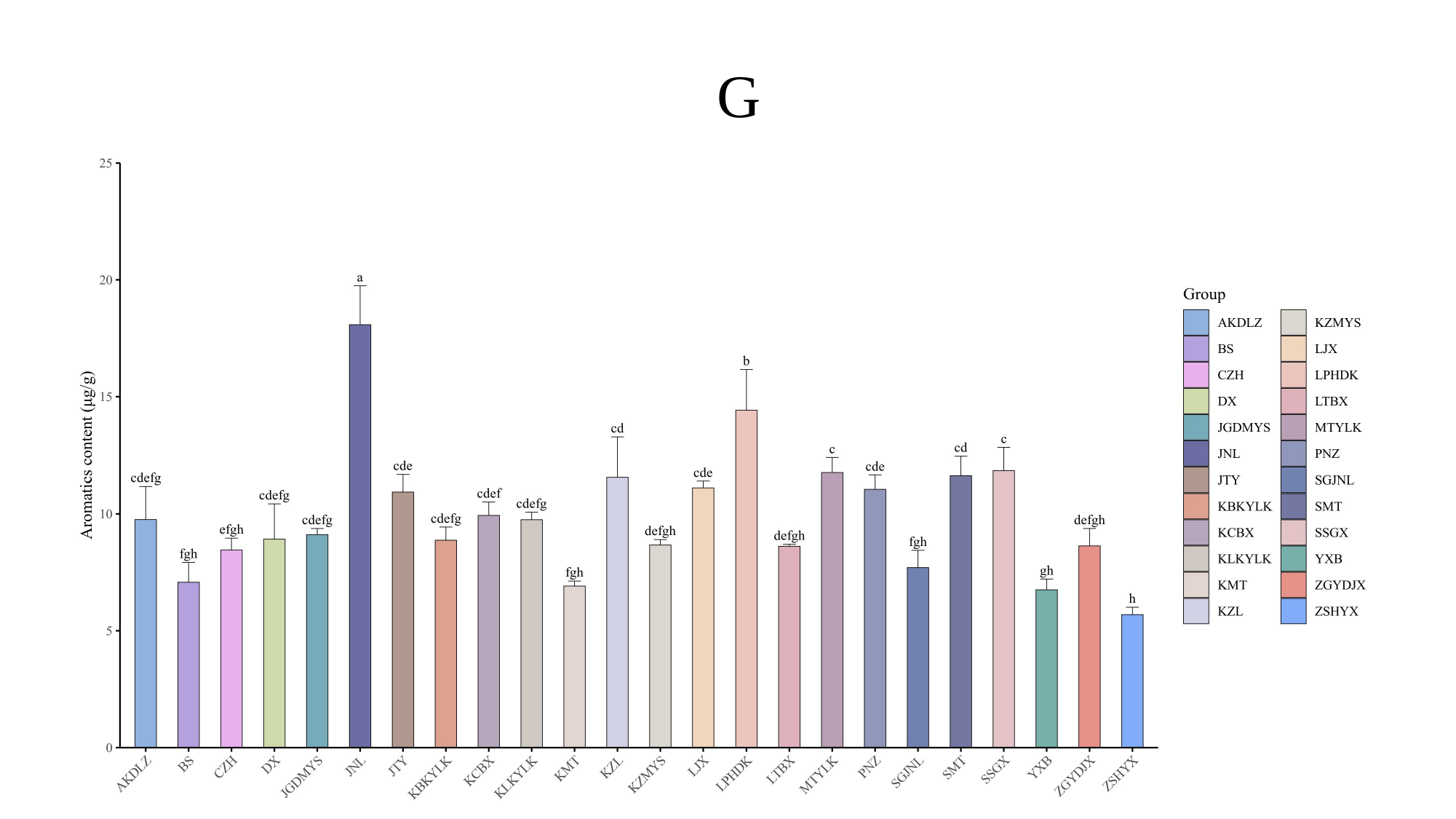

# G

## Slide 8
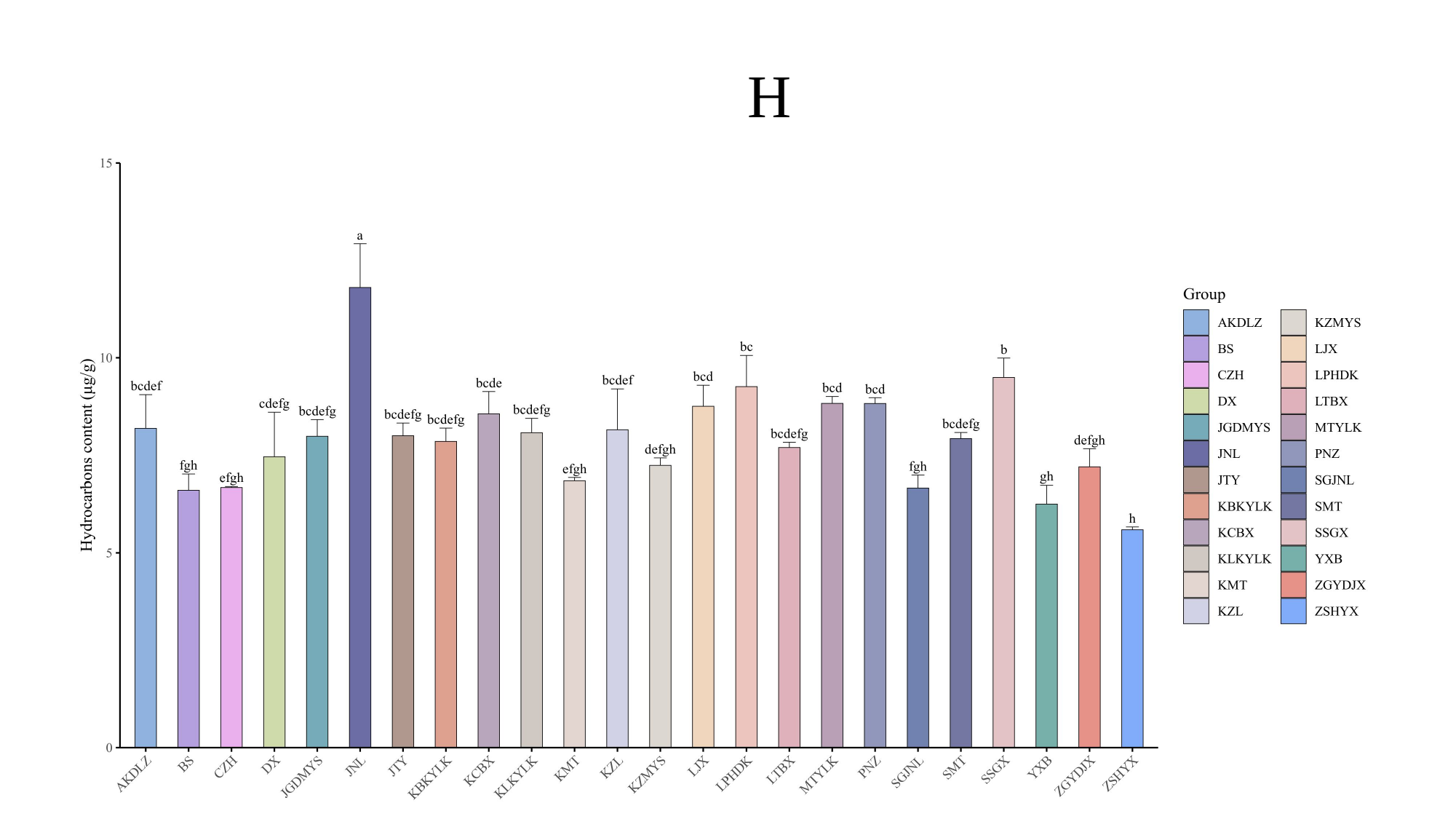

# H

## Slide 9
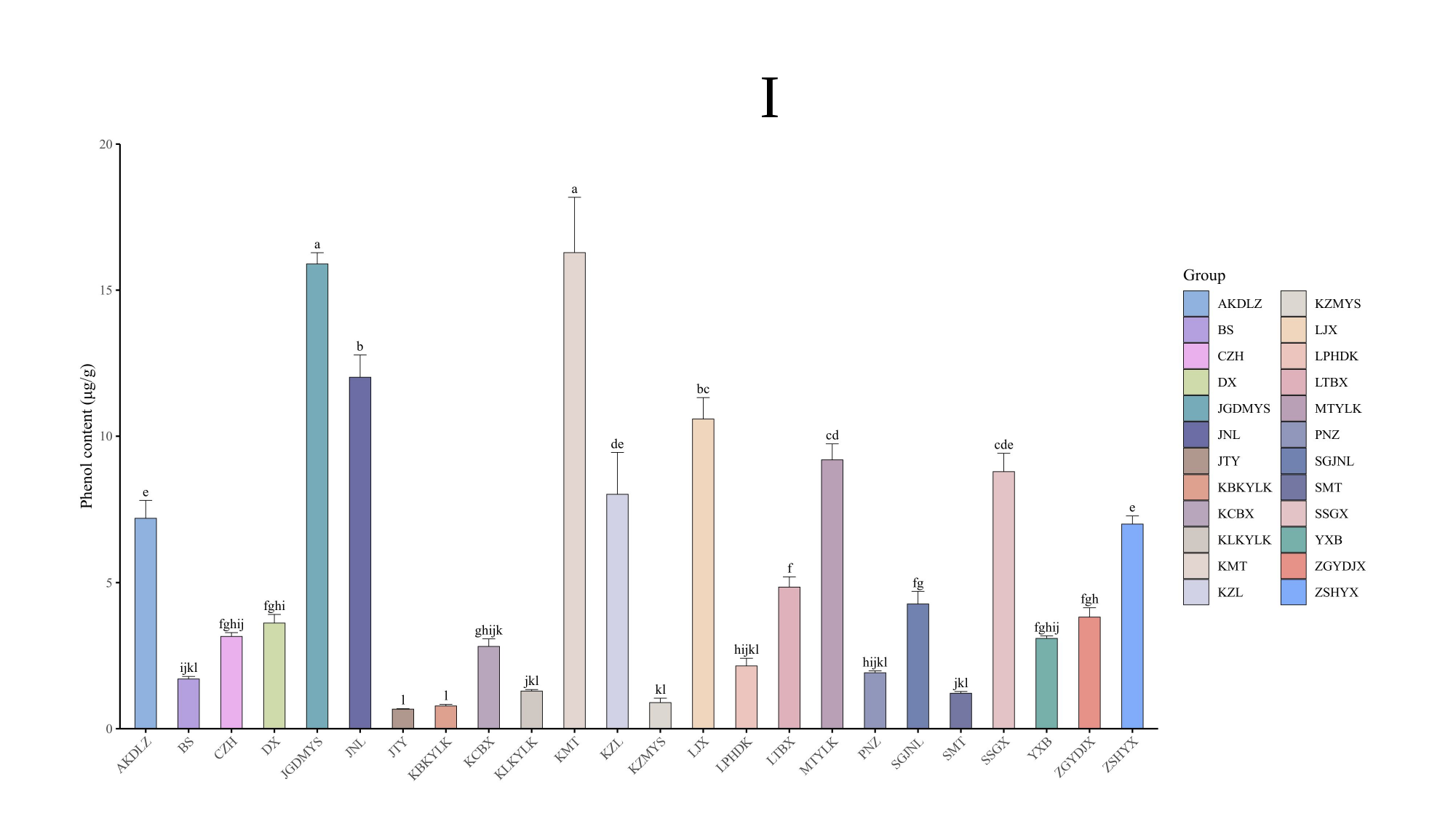

# I

## Slide 10
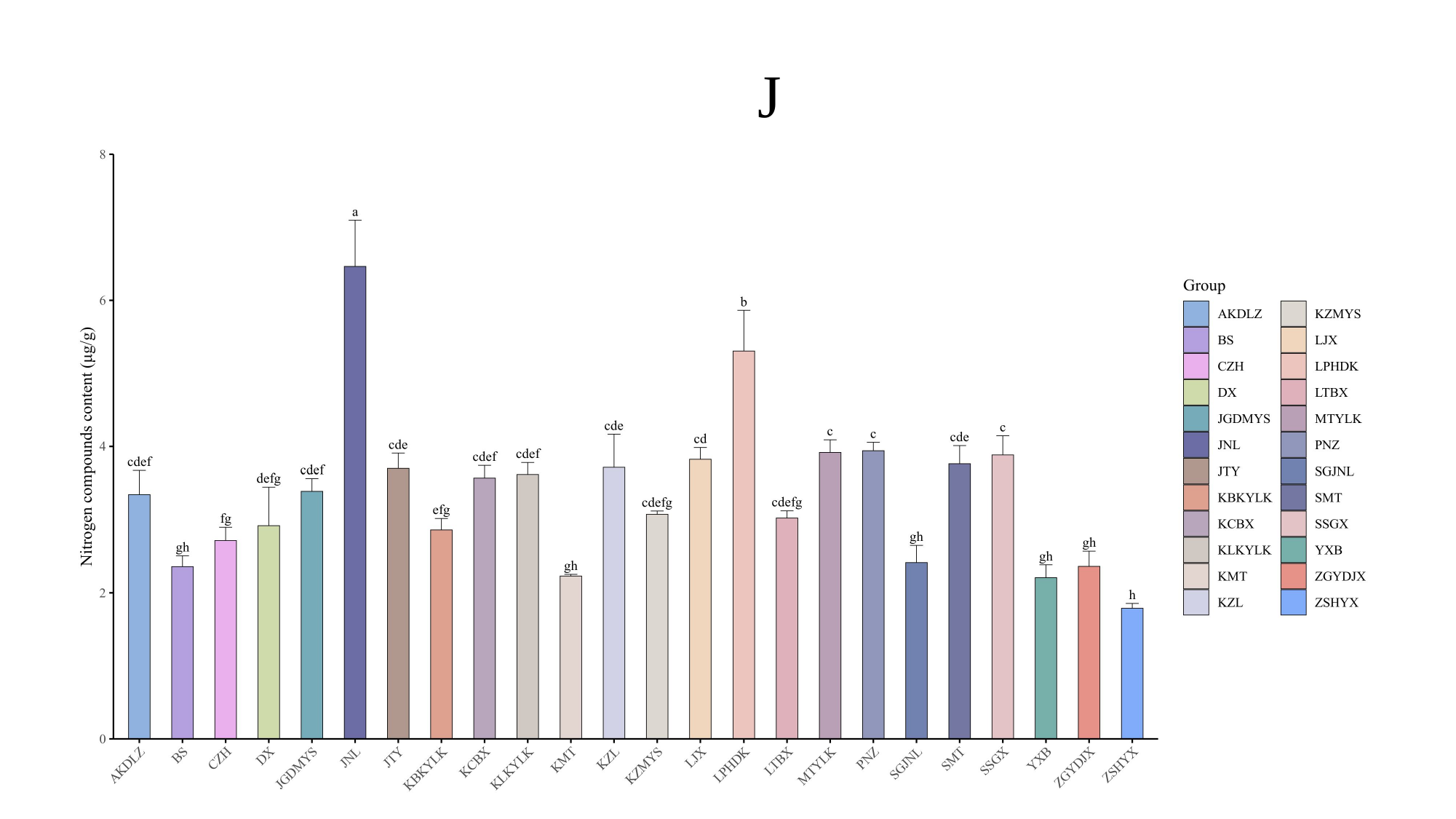

# J

## Slide 11
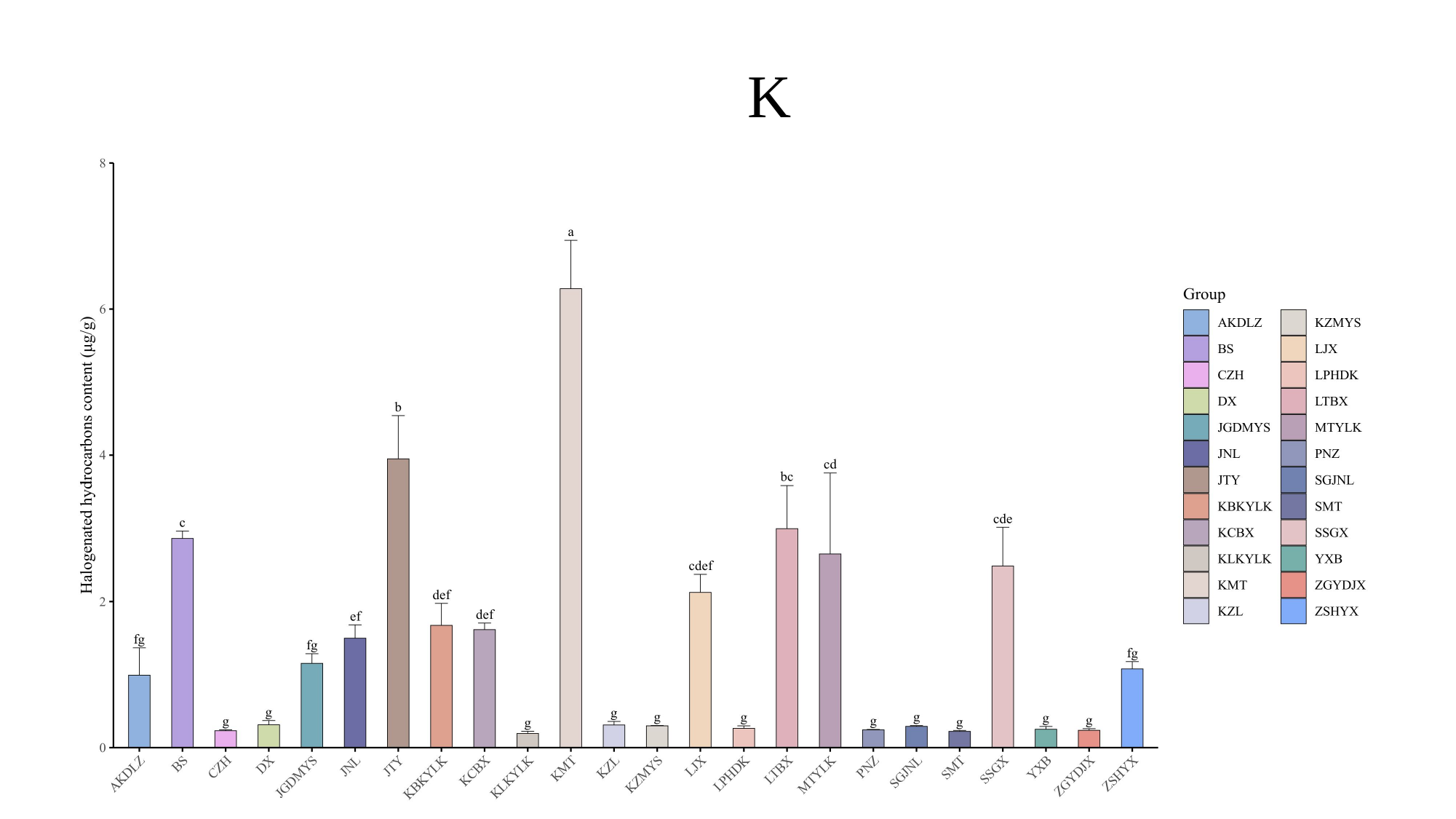

# K

## Slide 12
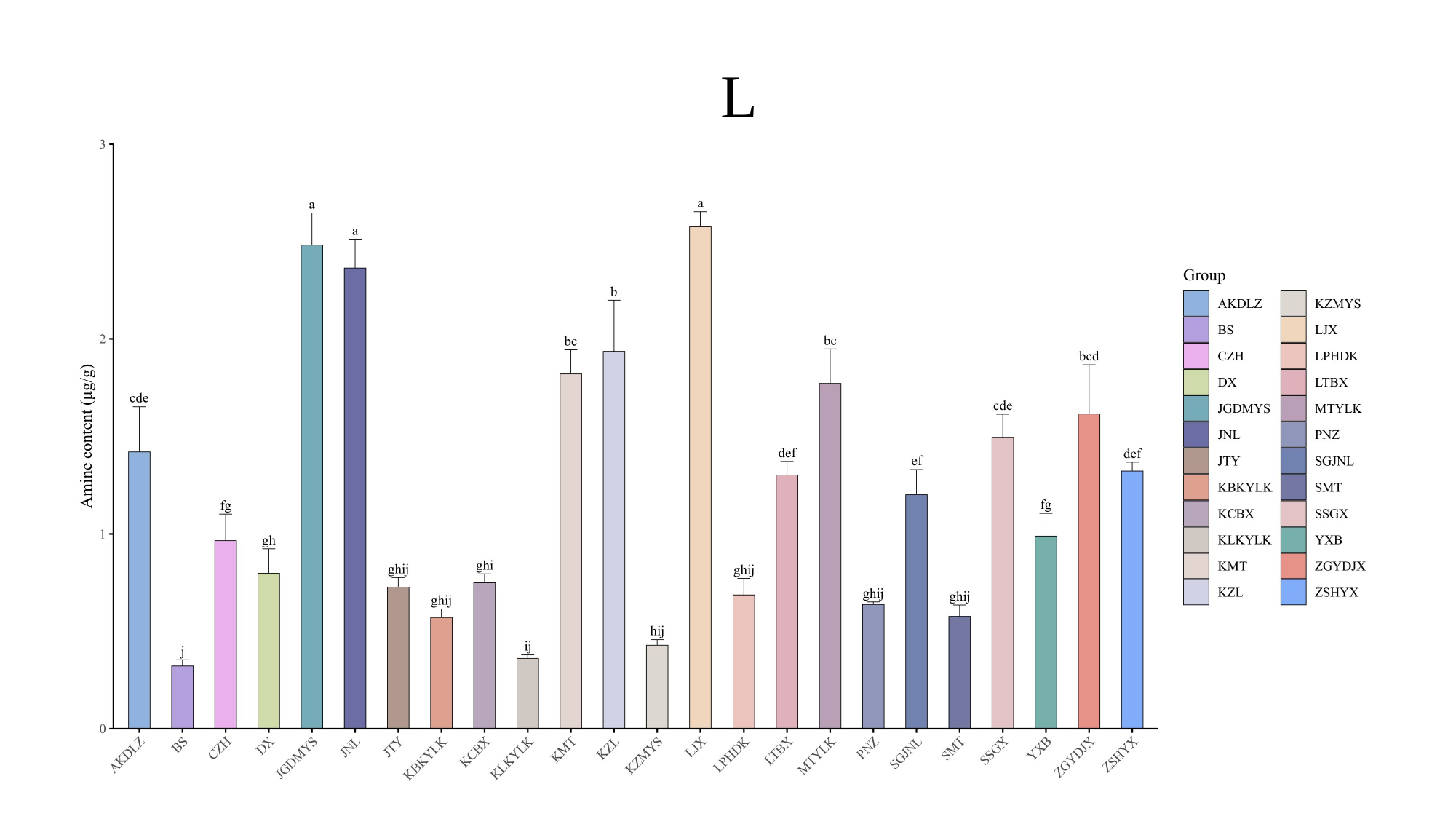

# L

## Slide 13
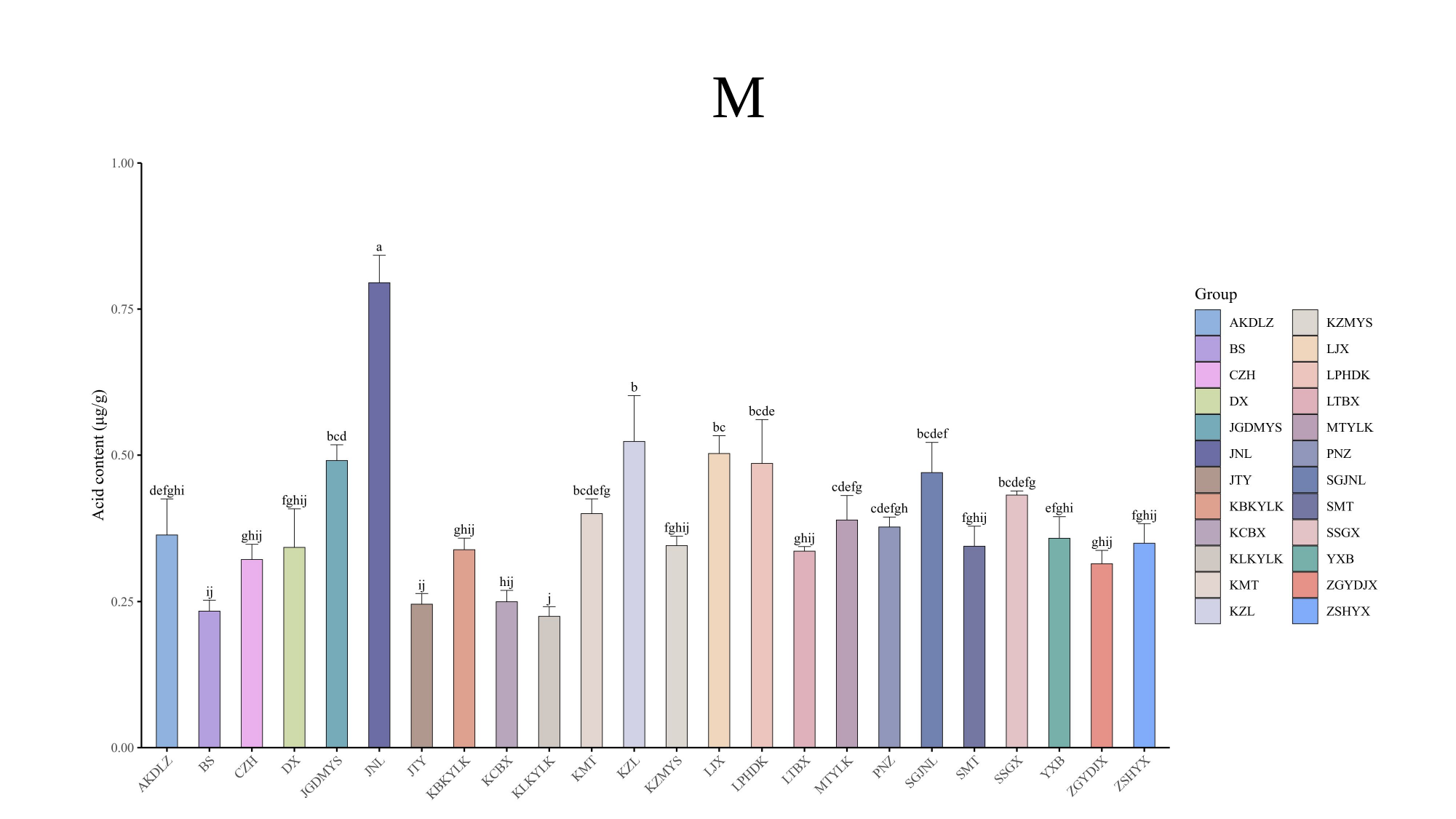

# M

## Slide 14
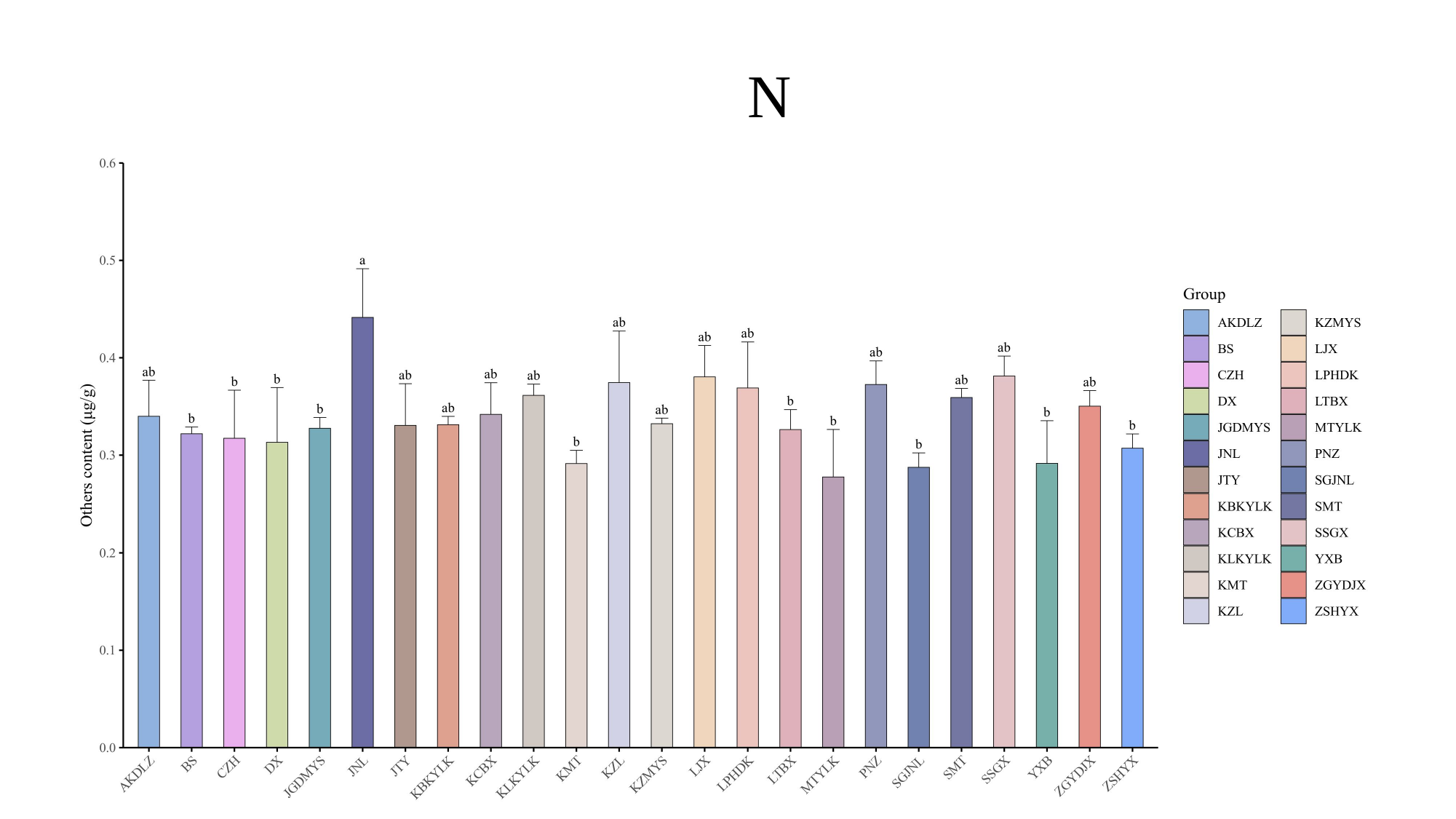

# N

## Slide 15
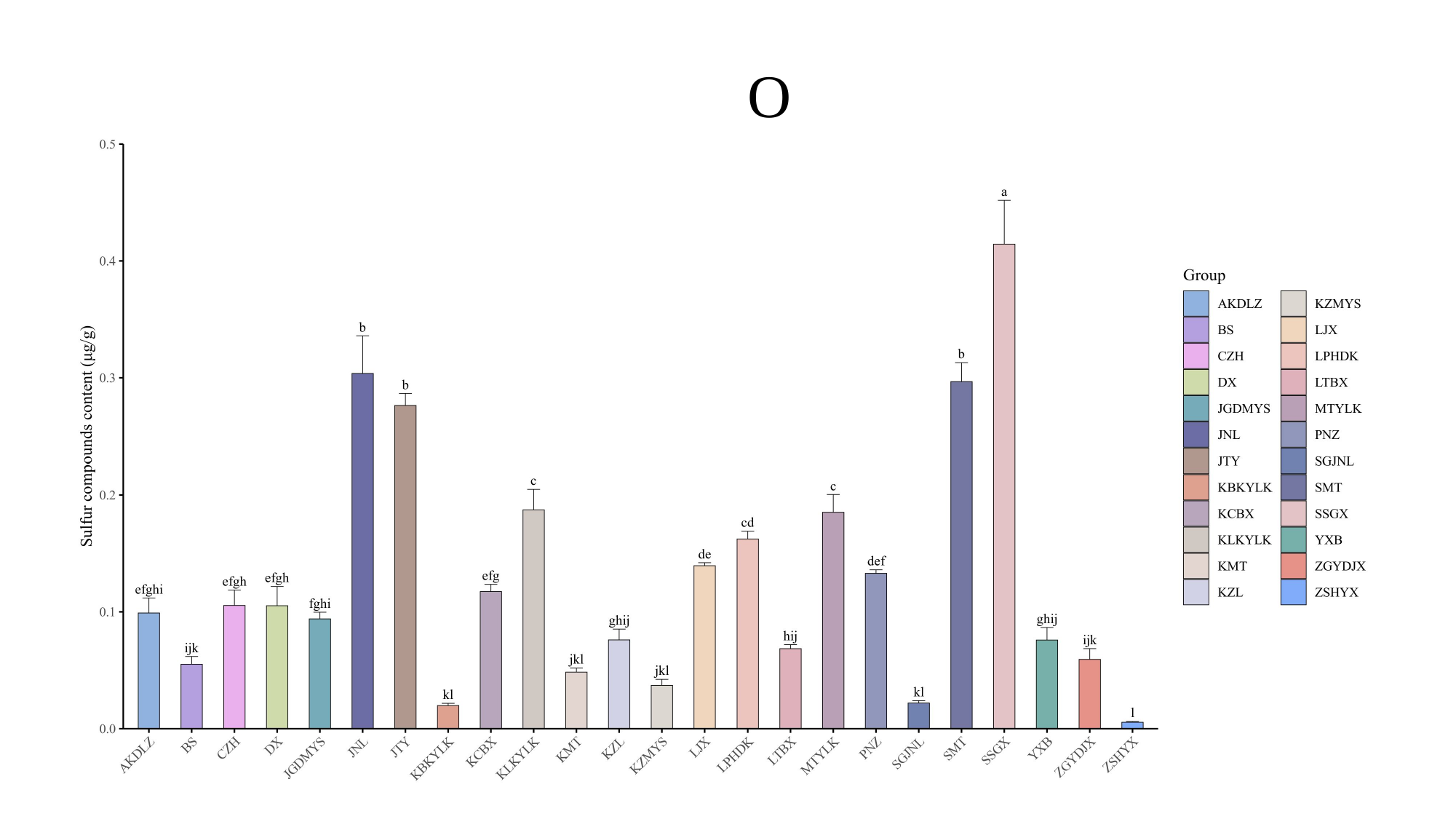

# O

## Slide 16
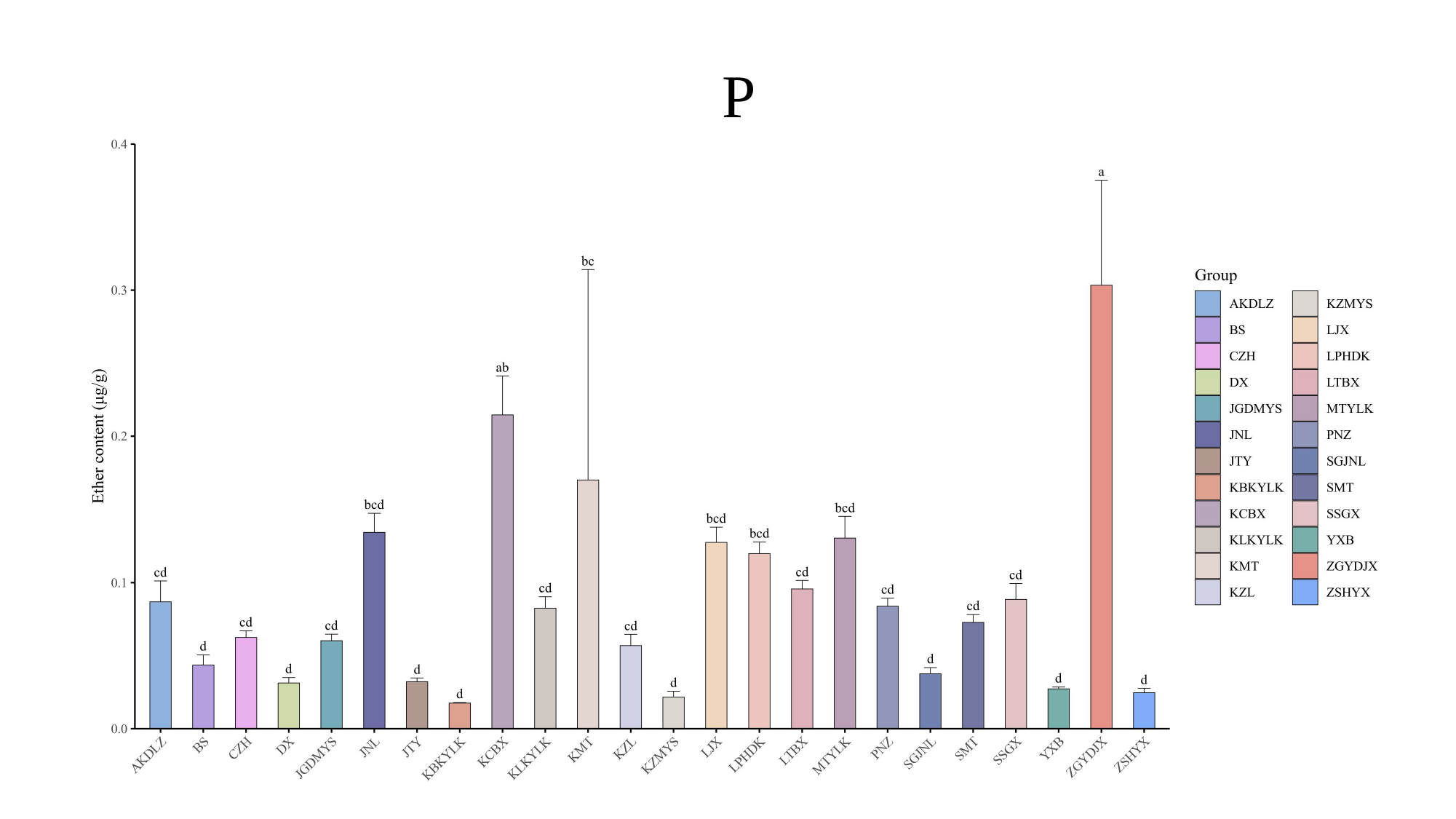

# P
