# Supplementary figures and images for: Headspace Solid-Phase Microextraction and Gas Chromatography–Mass Spectrometry Combined with Sensory Evaluation for the Analysis of Volatile Aromatic Compounds in Apricot (Prunus armeniaca L.) Germplasm Resources Cultivated in Xinjiang, China
Source: Foods. 2024 Dec 3;13(23):3912. doi: 10.3390/foods13233912 (PMC11640270; doi:10.3390/foods13233912)

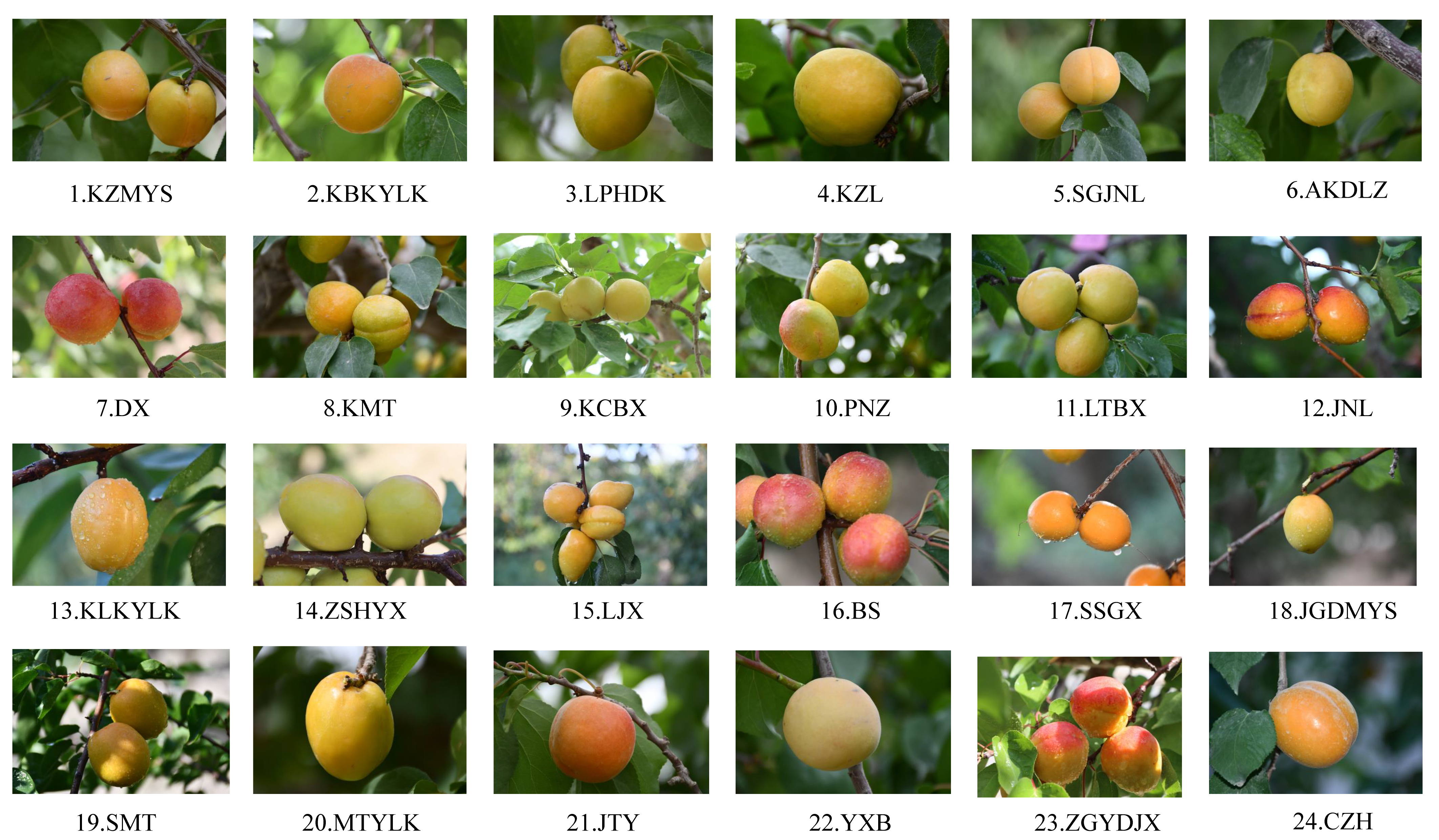

Supplement: Supplementary file 1 [file foods-13-03912-s001.zip › Supplementary Figure S1.tif]
